# Li/Al‐LDH Reinforced Polyacrylamide/Xanthan Gum Semi‐Interpenetrating Network Nano‐Conductive Hydrogels for Stress Sensing and Wearable Device Applications

**DOI:** 10.1002/advs.202511903

**Published:** 2025-09-30

**Authors:** Zhiwei Hu, Tuo Li, Yong Zheng, Shengxi Chen, Tong Wan, Hamdy Khamees Thabet, Zeinhom M. El‐Bahy, Dalal A. Alshammari, Hanhui Lei, Liqiang Chu, Yunlong Sun, Yaohui Guo, Yizhou Yang, Terence Xiaoteng Liu, Dapeng Cui, Zhanhu Guo, Huige Wei

**Affiliations:** ^1^ State Key Laboratory of Bio‐based Fiber Materials Tianjin Key Laboratory of Brine Chemical Engineering and Resource Eco‐Utilization College of Chemical Engineering and Materials Science Tianjin University of Science and Technology Tianjin 300457 China; ^2^ Center for Scientific Research and Entrepreneurship Northern Border University Arar 73213 Saudi Arabia; ^3^ Department of Chemistry Faculty of Science Al‐Azhar University Nasr City Cairo 11884 Egypt; ^4^ Department of Chemistry College of Science University of Hafr Al Batin Hafr Al Batin 39524 Saudi Arabia; ^5^ Faculty of Engineering and Environment Northumbria University Newcastle Upon Tyne NE1 8ST UK; ^6^ College of Light Industry Science and Engineering Tianjin University of Science and Technology Tianjin 300457 China; ^7^ College of Electronic Information and Automation Tianjin University of Science and Technology Tianjin 300457 China; ^8^ Department of Mechanical and Construction Engineering Northumbria University Newcastle Upon Tyne NE1 8ST UK

**Keywords:** conductive hydrogels, flexible wearable electronics, in situ polymerization, Li/Al‐LDH

## Abstract

Layered double hydroxides (LDHs) have gained significant attention for their unique physicochemical properties, but their application in conductive hydrogels for strain‐sensing still remains rarely explored due to their low electrical conductivity and poor compatibility with the hydrogel network. This study proposes an innovative strategy of preparing highly conductive and mechanically robust Li/Al‐LDH reinforced polyacrylamide (PAM)/xanthan gum (XG) semi‐interpenetrating network nano‐conductive hydrogels (PXL) by in situ polymerization of acrylamide (AM) monomers in Li/Al‐LDH colloidal solution. Li/Al‐LDH exhibits high electrical conductivity and meanwhile interacts with the polymer matrix to form coordination/hydrogen bonds. The unique multi‐collaborative network endows the PXL hydrogel with excellent mechanical properties (the strain at break is 2350%) and high sensing properties (the gauge factor is 4.65). As a proof of concept, an 8 × 8 sensor array and an intelligent insole are designed based on the PXL hydrogel, demonstrating the great broad prospects of PXL in medical, human‐computer interaction, and flexible wearable applications. This study provides new insights for introducing highly conductive and uniformly dispersed LDHs into hydrogels for flexible wearable electronics.

## Graphical abstract

PXL hydrogel is prepared by in situ polymerization of acrylamide (AM) between Li/Al‐LDH nanosheets and forming a semi‐interpenetrating network structure with xanthan gum (XG). The in situ polymerization of AM ensures the uniform distribution of Li/Al‐LDH within the hydrogel network. This multi‐collaborative network endows hydrogel with excellent mechanical and electromechanical properties, demonstrating great potential for applications in flexible electronics.

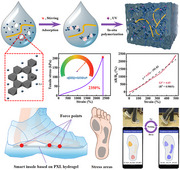

## 1 Introduction

With the arrival of the era of intelligent electronics,^[^ ^1^, ^2^ ^]^ flexible electronic devices have received extensive attention.^[^ ^3^, ^4^ ^]^ As the core of flexible electronic devices, flexible electronic materials are expected to become the “bearing” that promotes the progress of flexible electronic technology. Conductive hydrogels are undoubtedly one of the leading roles in the field of flexible electronic materials^[^ ^5^ ^]^ due to their excellent flexibility,^[^ ^6^ ^]^ biocompatibility,^[^ ^7^ ^]^ and high conductivity.^[^ ^8^ ^]^ The unique internal structure of conductive hydrogels can convert the detected external forces and deformations into recordable electrical signals,^[^ ^9^ ^]^ which endows them with extremely broad application prospects, such as sensors, medical devices, energy conversion, and storage devices.^[^ ^10^, ^11^ ^]^

In the preparation of conductive hydrogels, polymer matrices such as polyacrylamide (PAM),^[^ ^12^ ^]^ polyvinyl alcohol (PVA)^[^ ^13^ ^]^ are typically integrated with conductive fillers, for example, 2D conductive materials (graphene,^[^ ^14^ ^]^ MXene^[^ ^15^, ^16^ ^]^), conductive polymers (polythiophene,^[^ ^17^ ^]^ polyaniline (PANI)^[^ ^18^, ^19^ ^]^), or free ions.^[^ ^20^, ^21^ ^]^ Particularly, PAM‐based hydrogels have garnered significant attention owing to their merits of simple synthesis, good hydrophilicity, and low‐cost.^[^ ^22^ ^]^ However, the mechanical properties of pure PAM‐based hydrogels are very poor, and often a second network such as xanthan gum (XG), sodium alginate (SA) needs to be introduced for the network structure design.^[^ ^23^, ^24^ ^]^ As a biocompatible natural polysaccharide with an ordered double helix chain structure, XG contains abundant ─COOH and ─OH along its side chains, providing a large number of binding sites for ion coordination interaction and hydrogen‐bond formation and therefore is of great help in improving the mechanical properties of the hydrogels.^[^ ^25^ ^]^

Nano‐conductive hydrogels^[^ ^26^ ^]^ enhance hydrogel matrices by incorporating rigid, conductive nanomaterials, thereby granting them exceptional electrical conductivity and mechanical properties.^[^ ^27^, ^28^ ^]^ Layered double hydroxides (LDHs), with their tunable layered structures and biocompatibility,^[^ ^29^ ^]^ have garnered increasing attention in recent years.^[^ ^30^, ^31^ ^]^ Composed of positively charged layers and interlayer anions that balance charges, LDHs with a general formula of *[M^2+^_1‐x_M^3+^_x_(OH)_2_]^x+^A^y−^_x/y_·mH_2_O* hold promise for sensing applications. However, their utilization as conductive fillers in hydrogels remains rarely explored, primarily due to two critical challenges: a relatively low intrinsic electrical conductivity^[^ ^32^ ^]^ and a pronounced tendency to stack, which hinders a uniform dispersion within hydrogel networks.^[^ ^33^ ^]^ To address these limitations, rationally regulating the types and proportions of metal ions on the layers has become an effective strategy to enhance electrical conductivity. Among LDHs, Li/Al‐LDH with the molecular formula *[LiAl_2_(OH)_6_]^+^A^y−^_1/y_·mH_2_O*, stands out due to its highest layer charge density^[^ ^34^ ^]^ and unique structural features: Li⁺ ions occupy octahedral vacancies within the Al(OH)_3_ layers, enabling rapid diffusion through intralayer or interlayer vacancies, thereby enhancing ionic conductivity. Notably, during the synthesis of LDHs by the urea decomposition method, Yang et al.^[^ ^35^ ^]^ optimized the size of LDHs nanosheets by monitoring the Tyndall effect. Subsequently, in situ polymerization of PAM was conducted between the LDHs nanosheets, which effectively suppressed their restacking and achieved uniform dispersion of LDHs. This approach successfully resulted in the preparation of Co/Al‐LDH/PAM nano‐hydrogel. By comparison, this facile and environmentally friendly approach, enabled the uniform dispersion of conductive fillers in the hydrogel while avoiding the complex operation of the template method^[^ ^36^ ^]^ and the pollution caused by chemical modification.^[^ ^37^ ^]^ Therefore, the possibility of directly constructing nano‐conductive hydrogels using freshly prepared Li/Al‐LDH solution with the Tyndall effect has important practical significance.

This paper presents a simple, green, and innovative strategy to prepare Li/Al‐LDH enhanced PAM/XG semi‐interpenetrating network nano‐conductive hydrogel of PXL (short for PAM/XG/LDHs, where LDHs specifically refer to Li/Al‐LDH in the present study). By in situ polymerizing acrylamide (AM) between Li/Al‐LDH nanosheets to form conductive pathways^[^ ^35^ ^]^ and using xanthan gum (XG) to construct a semi‐interpenetrating network structure, the electrical and mechanical properties of the hydrogel are significantly improved. Through molecular dynamics simulations, the reinforcing effect of XG and Li/Al‐LDH nanosheets on the mechanical properties of hydrogels is investigated. The unique multi‐collaborative network endows the PXL hydrogel with excellent mechanical properties (experimental tests demonstrated a fracture strain reaching 2350% and a fracture tensile strength of up to 208 kPa). The PXL hydrogel is used for stress‐sensing applications and has a high tensile sensitivity (*GF* = 4.65), long‐term durability of more than 3600 consecutive cycles. Due to its excellent sensitivity, various human body motion signals can be detected.^[^ ^38^, ^39^ ^]^ Finally, with PXL hydrogel as the core sensing material, practical applications such as the 8 × 8 array sensor and the three‐point plantar pressure‐sensing smart insole have also been constructed, demonstrating the great broad prospects of PXL hydrogel in medical,^[^ ^40^ ^]^ human‐computer interaction,^[^ ^41^ ^]^ and flexible wearable applications.^[^ ^42^, ^43^ ^]^

## 2 Results and Discussion

### 2.1 Preparation of PXL Hydrogel

**Figure 1:**
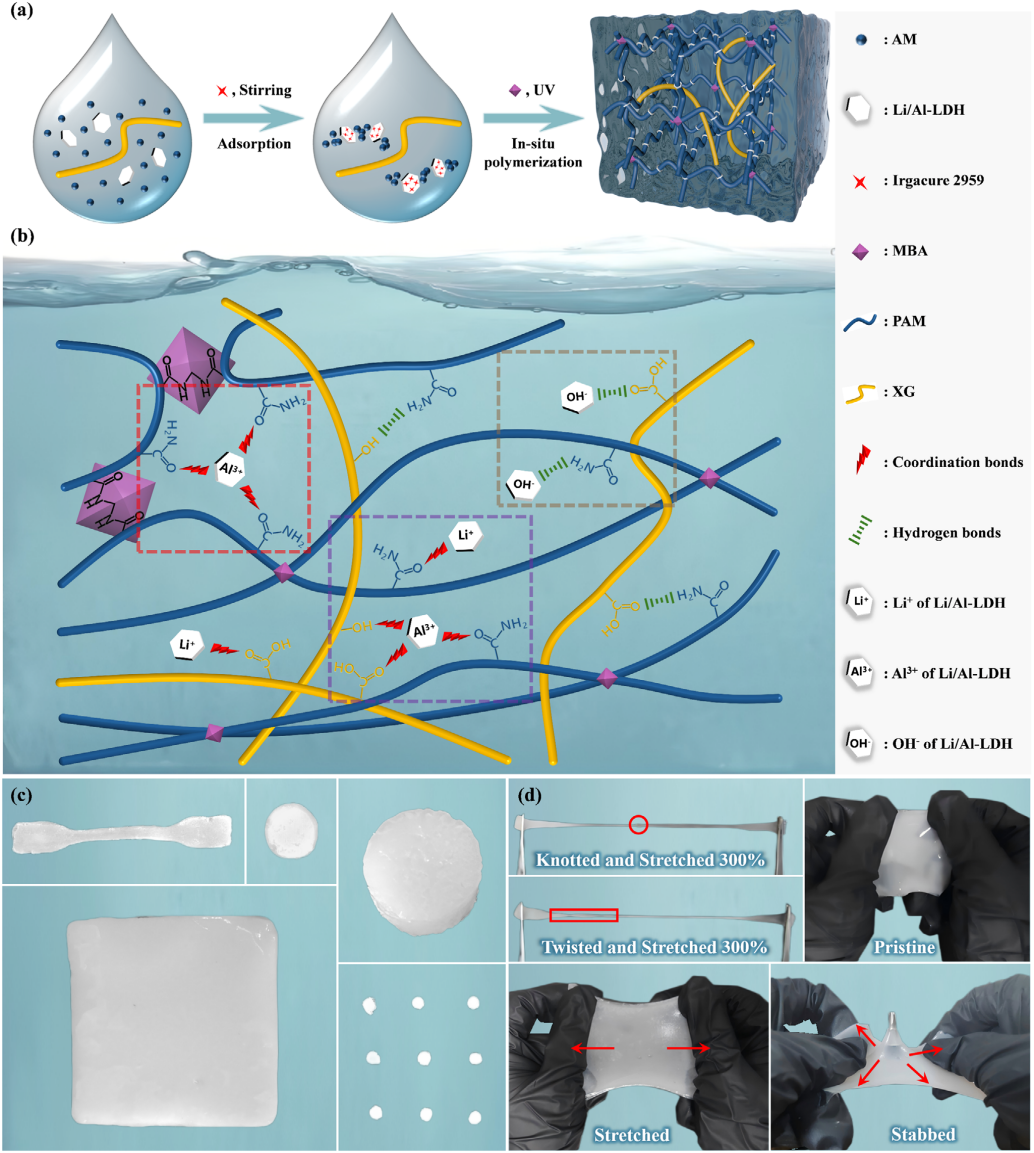
Hydrogel network and photographs of PXL nano‐conductive hydrogel. a) Preparation route of PXL. b) Schematic diagram of the PXL network structure. c) Digital photographs of PXL hydrogels of different shapes. Digital photographs showing d) knotted‐tensioned and twisted‐tensioned PXL splines and pristine, stretched, and stabbed PXL films.

The PXL nano‐conductive hydrogel was prepared by a two‐step method. First, a Li/Al‐LDH solution with a Tyndall effect was prepared. The structure of Li/Al‐LDH is illustrated in Figure S1 (Supporting Information). Then, AM monomers were in situ polymerized between Li/Al‐LDH nanosheets by a one‐pot method to obtain PXL nano‐conductive hydrogel (Figure S2, Supporting Information). As shown in Figure S3a (Supporting Information), when a red laser pointer was used to irradiate the Li/Al‐LDH solution reacting for 2 h, a bright red‐light path was formed in the solution, with an obvious Tyndall effect. This indicates that the size of Li/Al‐LDH nanosheets is between 1 and 100 nm, and they are uniformly distributed in the solution, which can be confirmed by the particle size distribution of Li/Al‐LDH (Figure S3b, Supporting Information). During the in situ polymerization of AM (**Figure** **1**a), Irgacure 2959, which serves as the initiator for PAM, is adsorbed on the surface by cations on the Li/Al‐LDH nanosheets due to adsorption.^[^ ^44^ ^]^ Under the ultraviolet irradiation, the initiator decomposes and forms primary free radicals. This confines the in situ polymerization of PAM macromolecular chains to the spaces between the nanosheets, thereby effectively preventing the re‐stacking of the nanosheets and achieving the uniform dispersion of Li/Al‐LDH nanosheets. The process continued until chain termination occurred, at which point the PAM network formed a semi‐interpenetrating network structure with XG long chains, ultimately yielding the PXL nano‐conductive hydrogels.

Regarding the internal microstructure of PXL (Figure 1b), PAM forms the main hydrophilic skeleton of the hydrogel and forms covalent bonds through MBA cross‐linking. Rigid XG, serving as the second network, interacts with PAM via hydrogen bonding to form a semi‐interpenetrating network structure. Li/Al‐LDH nanosheets, as reinforcing agents, the metal cations on the sheet surface can cause various coordination effects to form coordination bonds, and the presence of Al^3+^ can also complex with ─OH. In addition, Li/Al‐LDH nanosheets also have a large number of OH^−^, which can form hydrogen‐bonding interactions with PAM and XG, greatly increasing the density of hydrogen bonds.^[^ ^45^ ^]^ In the microstructural diagram, the functions of the Li/Al‐LDH nanosheets are also further elaborated in detail. The red box indicates that a coordination bond is formed between Al^3+^ on the Li/Al‐LDH nanosheet and the ─C(O)NH_2_ on the PAM chain, which is a weak complexation.^[^ ^46^ ^]^ This weak complexation effect constructs a 3D network structure of Li/Al‐LDH nanosheets cross‐linked with PAM. The purple box shows that Al^3+^ not only forms a coordination bond with the −C(O)NH_2_ on the PAM chain but also forms a coordination bond with the ─OH and ─COOH on the XG chain. Like a “bridge,” it further enhances the connection between PAM and XG. In addition, both Al^3^⁺ and Li⁺ simultaneously form coordination bonds with the functional groups on the PAM and XG chains. This bimetallic coordination can enhance the charge transfer ability of the hydrogel, thereby constructing abundant conductive pathways.^[^ ^47^ ^]^ As shown in the brown box, the OH^−^ on Li/Al‐LDH nanosheets form numerous hydrogen bonds with the groups on the PAM and XG chains. These numerous reversible sacrificial bonds have a significant impact on the mechanical properties of the entire hydrogel network. These milky white PXL hydrogels were obtained through in situ polymerization using different‐shaped molds (Figure 1c). The uniform color of the PXL hydrogel proves that there are uniformly dispersed Li/Al‐LDH nanosheets in the gel network. The synergistic effect of diverse components imparts the PXL hydrogel with superior mechanical properties, enabling it to withstand various deformations and stretches, such as knotting and twisting stretches up to 300%. Appropriate cross‐linking can endow the hydrogel film with excellent puncture resistance and ductility (Figure 1d). In addition, the presence of Li/Al‐LDH in the PXL hydrogel network endows the hydrogel with excellent electrical conductivity (Figure S4, Supporting Information), reaching 2.08 S m^−1^ at 25 °C. To determine the conduction mechanism of the PXL hydrogel, variable‐temperature conductivity tests were performed in the temperature range of 5–60 °C, and the results were plotted as an Arrhenius diagram (Figure S5a, Supporting Information). The Arrhenius diagram exhibits a linear relationship, and the activation energy (*E*_a_ = 0.35 eV) calculated from the slope of the straight line is relatively low. This confirms that the conductivity of the PXL hydrogel is almost entirely dominated by an ionic migration mechanism. The migration of Li^+^ ions in the hydration network acts as the main current carrier; the Li/Al‐LDH nanosheets primarily function as a structural scaffold and provide ion channels, with their own contribution to electronic conductivity being negligible. Additionally, EIS measurements of the PXL hydrogel were conducted at 25 °C (Figure S5b, Supporting Information). Through equivalent circuit fitting and calculation, the ionic conductivity (2.03 S m^−1^) of the PXL hydrogel is approximately equal to its total conductivity (2.08 S m^−1^), verifying that the current in the system is almost entirely contributed by the migration of Li^+^ ions.

To evaluate the water retention capacity, PAM, PX, and PXL hydrogels were exposed to 25 °C for 7 consecutive days, with their weight changes monitored in real‐time (Figure S6a, Supporting Information). The PAM hydrogel lost ≈80% of its initial weight within 1 day, whereas the PX hydrogel only lost ≈50% of its mass under the same conditions. This improvement is attributed to the formation of a semi‐interpenetrating network between PAM and XG, which effectively encapsulates water within a 3D structure, thereby restricting its free diffusion. The introduction of Li/Al‐LDH further enhanced water retention, with the PXL hydrogel retaining ≈65% of its initial mass after 7 days. This is primarily attributed to the presence of Li^+^ and Cl^−^, which reduces the vapor pressure of the hydrogel,^[^ ^48^ ^]^ and Al^3+^, forming coordination bonds and complexation with the ─OH groups in the polymer network through multiple coordination effects. This enhances the cross‐linking density of the hydrogel network, resulting in a denser and more stable 3D structure that more effectively traps water molecules within the network.^[^ ^49^ ^]^ Consequently, it further restricts the free diffusion and evaporation of moisture, enabling the hydrogel to withstand dry environments. As shown in Figure S6b (Supporting Information), the PAM hydrogel exhibits severe deformation and a loss of flexibility, while the PX hydrogel retains partial shape but reduces flexibility. In contrast, the PXL hydrogel maintains its original morphology and mechanical integrity, demonstrating excellent flexibility and resilience under external stress (Figure S6c, Supporting Information).

### 2.2 Characterizations of PXL Hydrogel

**Figure 2:**
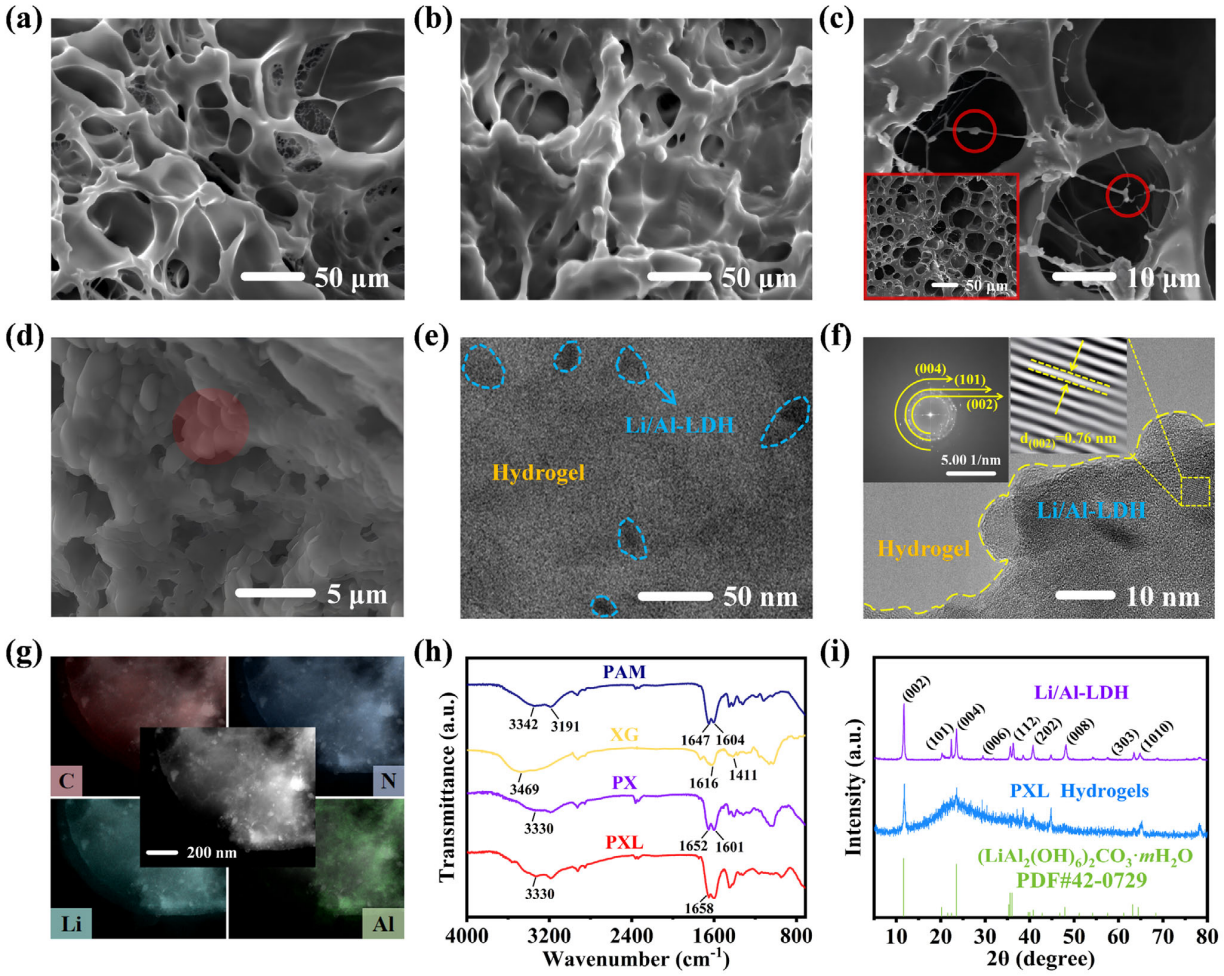
Cross‐sectional SEM images of a) PAM, b) PX, and c) PXL. d) SEM image of PXL surface (the “fish scale” structure is highlighted). e) TEM image of PXL at a low magnification. f) TEM image of PXL at a high magnification (with illustrations of the in situ SAED and lattice spacing). g) Elemental mappings of C, N, Li, and Al elements of PXL. h) Infrared spectra of PAM, XG, PX, and PXL. i) XRD patterns of Li/Al‐LDH and PXL.

The microstructures of PAM, PX, and PXL hydrogels were investigated by SEM images. The pure PAM hydrogel (**Figure** **2**a) exhibits a porous structure with smooth and thick walls, attributed to their high hydrogen‐bond density and dense packing of molecular chains.^[^ ^22^ ^]^ The introduction of XG results in a thicker pore wall in the PX hydrogel (Figure 2b), likely due to the penetration and entanglement of the XG network with the PAM network, which enhances the compactness of the structure. Further incorporation of Li/Al‐LDH leads to a significant increase in the pore density (Figure 2c) and the formation of a highly cross‐linked structure, which is beneficial for enhancing mechanical strength.^[^ ^50^, ^51^ ^]^ Additionally, fibrous structures within the pores that may form a conductive network are observed, enhancing both mechanical and electrical properties.^[^ ^52^ ^]^ Notably, the layered Li/Al‐LDH nanosheets are uniformly distributed on the surface and embedded within the PXL hydrogel, forming a regular “fish‐scale” structure (Figure 2d), indicating successful in situ polymerization of AM between the nanosheets. This uniform dispersion of Li/Al‐LDH contributes to the enhanced mechanical properties and electrical stability of the PXL hydrogel. Subsequently, the orientation angles of over 100 Li/Al‐LDH nanosheets in multiple regions were measured using ImageJ software. The average orientation degree was 48.3° (with a standard deviation of 2.6°), which indicates that the nanosheets exhibit a significant preferential orientation arrangement. This structure may contribute to anisotropic mechanical and electrical properties.

Subsequently, TEM analysis was performed to characterize the microstructure of the PXL hydrogel. Figure 2e reveals that the Li/Al‐LDH nanosheets are uniformly distributed within the hydrogel, and its size is consistent with the particle size analysis. At a higher magnification (Figure 2f), the in situ SAED pattern confirms the crystallinity of *(LiAl_2_(OH)_6_)_2_CO_3_·mH_2_O* in the PXL hydrogel, with the lattice fringe corresponding to the (002) crystal plane (*d* = 0.76 nm), indicating the successful embedding of Li/Al‐LDH without structural distortion.^[^ ^53^ ^]^ The elemental mapping (Figure 2g) further demonstrates the uniform distribution of C, N, Li, and Al, confirming the formation of a semi‐interpenetrating network.^[^ ^54^ ^]^ This structure endows the hydrogel with excellent anti‐swelling properties. After 7 days in deionized water, the PXL hydrogel exhibits a low swelling rate of 16.4% (Figure S7a, Supporting Information). In contrast, the swelling rate of the PXL hydrogel in physiological saline is even lower, at only 10% (Figure S7b, Supporting Information). This indicates that when the PXL hydrogel is applied to human multifunctional sensors, it can maintain shape stability on human skin for a long time, thereby ensuring the accuracy and continuous stability of sensing signals.

In order to study whether the prepared PXL hydrogel has introduced corresponding functional groups for cross‐linking, FTIR analysis was performed on these three hydrogels. Figure 2h shows the infrared spectrum of PAM, XG, PX, and PXL hydrogels. For the PAM hydrogel, the absorption peaks at 3342 and 3191 cm^−1^ correspond to the asymmetric and symmetric stretching vibrations of ─NH_2_ respectively.^[^ ^55^ ^]^ The bands at 1647 and 1604 cm^−1^ correspond to the C═O stretching vibration and the N─H bending vibration in the ─C(O)NH_2_, respectively.^[^ ^56^, ^57^ ^]^ For the XG, the absorption band at 3469 cm^−1^ in XG is attributed to the O─H stretching vibration. The characteristic peak at 1616 cm^−1^ is indicative of the C═O stretching vibration of the ─COOH on the pyruvate group, while the absorption peak at 1411 cm^−1^ is due to the symmetric stretching motion of the ─COO^−^ on the glucuronide.^[^ ^58^ ^]^ In the PX hydrogel, no new absorption peaks are observed compared to PAM and XG, indicating no chemical reaction between them. However, it can be observed that the stretching vibration absorption peak of the ─OH of the PX hydrogel shifts to 3330 cm^−1^. This is due to the formation of new intermolecular hydrogen bonds between XG and PAM.^[^ ^59^ ^]^ After adding Li/Al‐LDH, due to the presence of Al^3^⁺ in Li/Al‐LDH, Al^3^⁺ can complex with ─OH, which leads to a reduction in the ─OH functional group and thus a reduction in the number of hydrogen bonds. Therefore, the ─OH stretching vibration band at 3330 cm^−1^ becomes narrower and weaker.^[^ ^53^ ^]^ In addition, the C═O stretching vibration absorption peak of XG in the PXL hydrogel changes between 1616 and 1658 cm^−1^. This is attributed to the formation of metal coordination bonds between ─COOH on XG and Li⁺ and Al^3^⁺ in LDH.^[^ ^60^ ^]^ XRD analysis further confirms the presence of Li/Al‐LDH in the PXL hydrogel. The XRD pattern of the prepared Li/Al‐LDH is well‐matched to the standard card of *(LiAl_2_(OH)_6_)_2_CO_3_·mH_2_O*, with no significant impurity peaks (Figure 2i). Although the peak intensity of PXL is slightly reduced, the main XRD peaks remain consistent with those of pure Li/Al‐LDH, confirming the successful incorporation of Li/Al‐LDH into the hydrogel.^[^ ^61^ ^]^

### 2.3 Mechanical Properties of PXL Hydrogel

#### 2.3.1 Mechanical Properties Simulation of PXL Hydrogel

**Figure 3:**
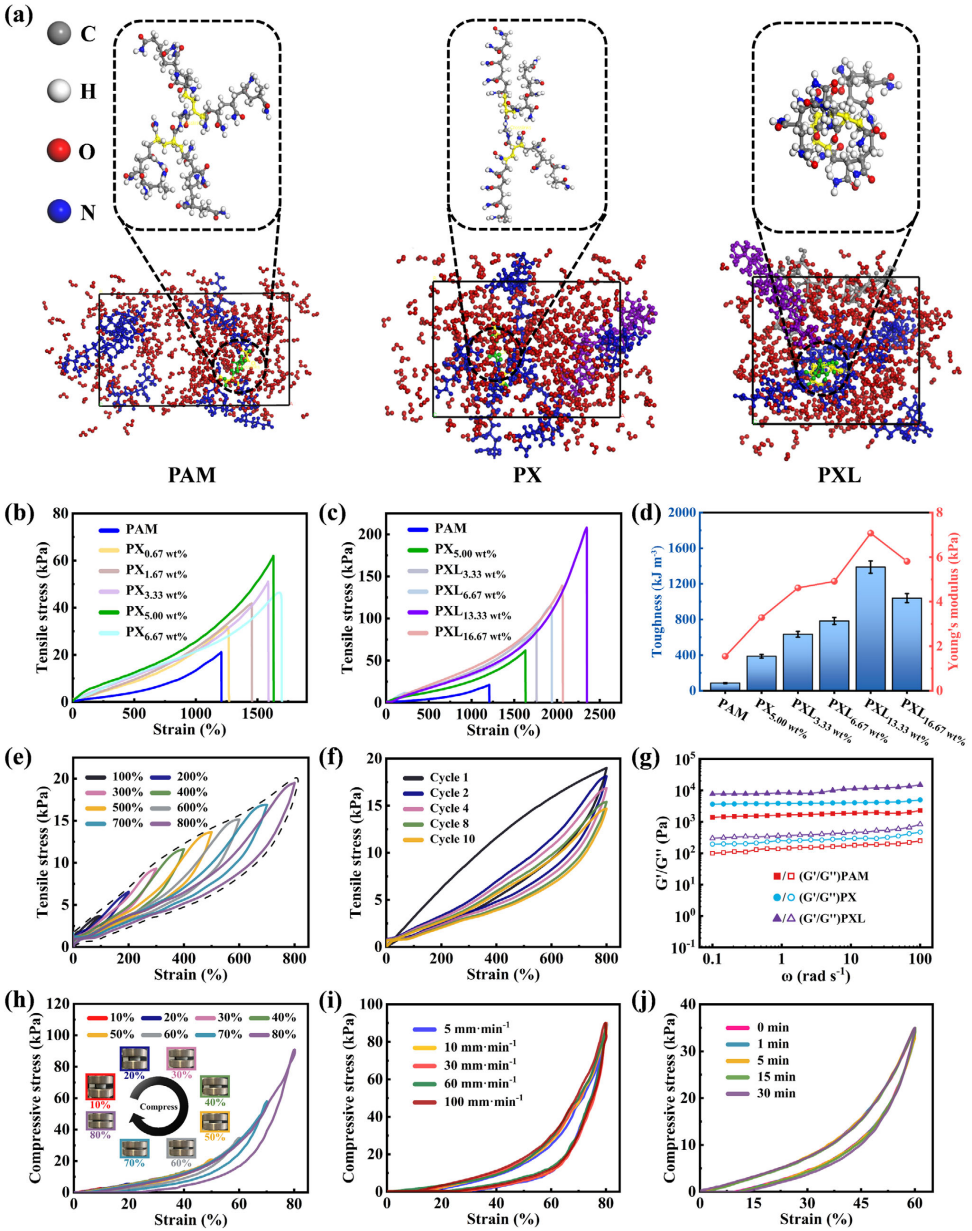
Mechanical properties of PXL hydrogel. a) The stress–strain simulation models of PAM, PX, and PXL hydrogels under a stress of 5 GPa (local magnification corresponds to the stretching state of cross‐linked parts). b) Tensile stress–strain curves of pure PAM hydrogel and PX hydrogels with different XG contents. c) Tensile stress–strain curves of PXL hydrogels with different Li/Al‐LDH contents. d) Toughness and Young's modulus of PXL hydrogels with different Li/Al‐LDH contents. e) Tensile stress–strain curves of PXL_13.33 wt.%_ hydrogel under increasing strain from 0 to 800%. f) Tensile stress–strain curves of PXL_13.33 wt.%_ hydrogel of ten cycles stretched to 800% without standing. g) *G'* and *G* *''* of PAM, PX_5.00 wt.%_ and PXL_13.33 wt.%_ hydrogels under increased angular frequency scanning (0.1–100 rad s^−1^). Cyclic compressive loading–unloading curves of PXL_13.33 wt.%_ under h) different strains (10–80% strain, @60 mm min^−1^), i) different rates (80% strain, @5–100 mm min^−1^), and j) different residence times (60% strain, @40 mm min^−1^).

Molecular dynamics simulations using Materials Studio were performed to investigate the mechanical properties of hydrogels. The cross‐linking and stress–strain behaviors of PAM, PX, and PXL hydrogels were simulated (Figure S8a–c, Supporting Information), aiming to explore the trend of enhanced mechanical properties of the hydrogels with the successive addition of XG and Li/Al‐LDH. Under a stress of 5 GPa (**Figure** **3**a), the strain of the hydrogel decreases significantly from 93% for PAM to 53% for PX and finally to 27% for PXL, indicating that the introduction of XG and Li/Al‐LDH progressively improves the mechanical properties of the hydrogel. The cross‐linking principle of the hydrogel is shown in Figure S9a (Supporting Information). The stress–strain curves (Figure S9b, Supporting Information) further confirm this trend, with the Young's modulus of PXL reaching ≈16 GPa, which is ≈3.5 times higher than that of PAM and two times higher than that of PX (Figure S9c, Supporting Information). These results demonstrate that the combination of XG and Li/Al‐LDH effectively enhances the mechanical properties of the hydrogel through cross‐linking and structural reinforcement.

#### 2.3.2 Tensile Mechanical Properties of PXL Hydrogel

Mechanical properties serve as the foundation for the practical implementation of hydrogels. The crux lies in regulating the subtle equilibrium between toughness and stiffness, while preserving a high tensile ratio.^[^ ^62^ ^]^ Figure 3b,c demonstrates the enhancement of mechanical properties in hydrogels by varying the amounts of XG and Li/Al‐LDH. For pure PAM hydrogel, the tensile elongation reaches 1200%, but its tensile strength is only 20 kPa. Upon the addition of XG, the tensile strength and strain of the hydrogel are improved. The PX_5.00 wt.%_ hydrogel exhibits a tensile strain of 1630% and a fracture strength of 60 kPa, demonstrating a good balance between rigidity and toughness. This improvement is attributed to the formation of a rigid‐flexible semi‐interpenetrating polymer network (SIPN) structure, where the rigid XG double helix chains interact with the flexible PAM chains, and hydrogen bonds between ─OH and ─COOH groups on XG and ─C(O)NH_2_ on PAM contribute to the enhanced mechanical stability.^[^ ^63^ ^]^ The incorporation of Li/Al‐LDH as nanofillers further enhances the mechanical properties. Among them, the PXL_13.33 wt.%_ hydrogel exhibits an optimal performance with a tensile elongation of 2350% and a tensile strength of 208 kPa. The Li/Al‐LDH nanosheets, rich in Li⁺, Al^3^⁺, and OH^−^, form coordination and hydrogen‐bonding interactions with functional groups in the SIPN, significantly improving the mechanical strength. The PXL_13.33 wt.%_ hydrogel demonstrates a toughness of 1388 kJ m^−3^ and a Young's modulus of 7 kPa, which is ≈3.8 times that of PAM and 2.2 times that of PX_5.00 wt.%_ (Figure 3d). These results confirm the effectiveness of combining XG and Li/Al‐LDH to enhance the mechanical properties of hydrogels. At the same time, the consistency of this trend also confirms the validity of the conclusions drawn from the MD simulations.

Subsequently, the energy dissipation mechanism of hydrogels was investigated. When the tensile strain gradually increases from 100% to 800% (Figure 3e), the stress–strain curves exhibit hysteresis loops, with the area enclosed representing the energy dissipated during the deformation. This energy dissipation arises from the destruction of chemical cross‐linking, disentanglement of macromolecular chains, and breaking of dynamic metal‐coordinated bonds and hydrogen bonds.^[^ ^64^, ^65^ ^]^ The overlapping regions between successive hysteresis loops indicate the immediate recovery of the hydrogel after unloading, which is attributed to the rapid reconstruction of reversible sacrificial bonds such as electrostatic interactions, hydrogen bonds, and coordination bonds. Reversible sacrificial bonds are prone to break preferentially to dissipate energy, thus improving the hydrogel's capacity to withstand external stresses.^[^ ^66^, ^67^ ^]^

The PXL_13.33 wt.%_ hydrogel demonstrates excellent fatigue resistance, maintaining stability after ten cycles of 800% tensile and 80% compressive deformation (Figure 3f; Figure S10, Supporting Information).^[^ ^68^ ^]^ Despite experiencing plastic deformation and microstructural changes (such as chain orientation adjustment and hydrogen bond disruption/reconstruction), the hydrogel still demonstrates low stress accumulation and maintains a high recovery ratio. Meanwhile, the rheological analysis test in Figure 3g shows that the *G'* of the PXL_13.33 wt.%_ gel is nearly an order of magnitude higher than the *G'* of pure PAM and PX_5.00 wt.%_, proving the elasticity of the PXL hydrogel. For the PXL_13.33 wt.%_ hydrogel, in the low and medium angular frequency range of 0.1–100 rad s^−1^, *G'* is always higher than *G''*, evidence of the typical mechanical characteristics of dynamic physical bonds.^[^ ^69^ ^]^

#### 2.3.3 Compressive Mechanical Properties of PXL Hydrogel

In addition, the PXL_13.33 wt.%_ cylindrical hydrogel undergoes cyclic compressive tests with gradually increasing compressive amounts from 10% to 80% (Figure 3h). It can even quickly recover to the original state upon the external force is removed when subjected to a high compressive deformation of up to 80% (Video S1, Supporting Information), indicating an excellent elastic range and a high elastic limit. Subsequently, the hydrogels were subjected to continuous compression cycles with 80% deformation at different rates (Figure 3i). The stress–strain curves of cyclic compression and unloading show a high degree of overlap, indicating that the hydrogel has a high elastic modulus. At different rates, the abundant dynamic physical crosslinks within the PXL hydrogel effectively resist deformation and rapidly recover, indicating its excellent toughness, which helps maintain structural stability under various external forces.^[^ ^70^ ^]^ In the cyclic compression tests at 60% strain, the PXL hydrogel consistently exhibits stable recovery after different compression dwell times (Figure 3j), demonstrating its internal structure's ability to effectively store and release energy. This further highlights the promising application potential of PXL hydrogel in flexible sensor devices.

### 2.4 Electromechanical Properties of PXL Hydrogel

#### 2.4.1 Tensile Electromechanical Properties of PXL Hydrogel

**Figure 4:**
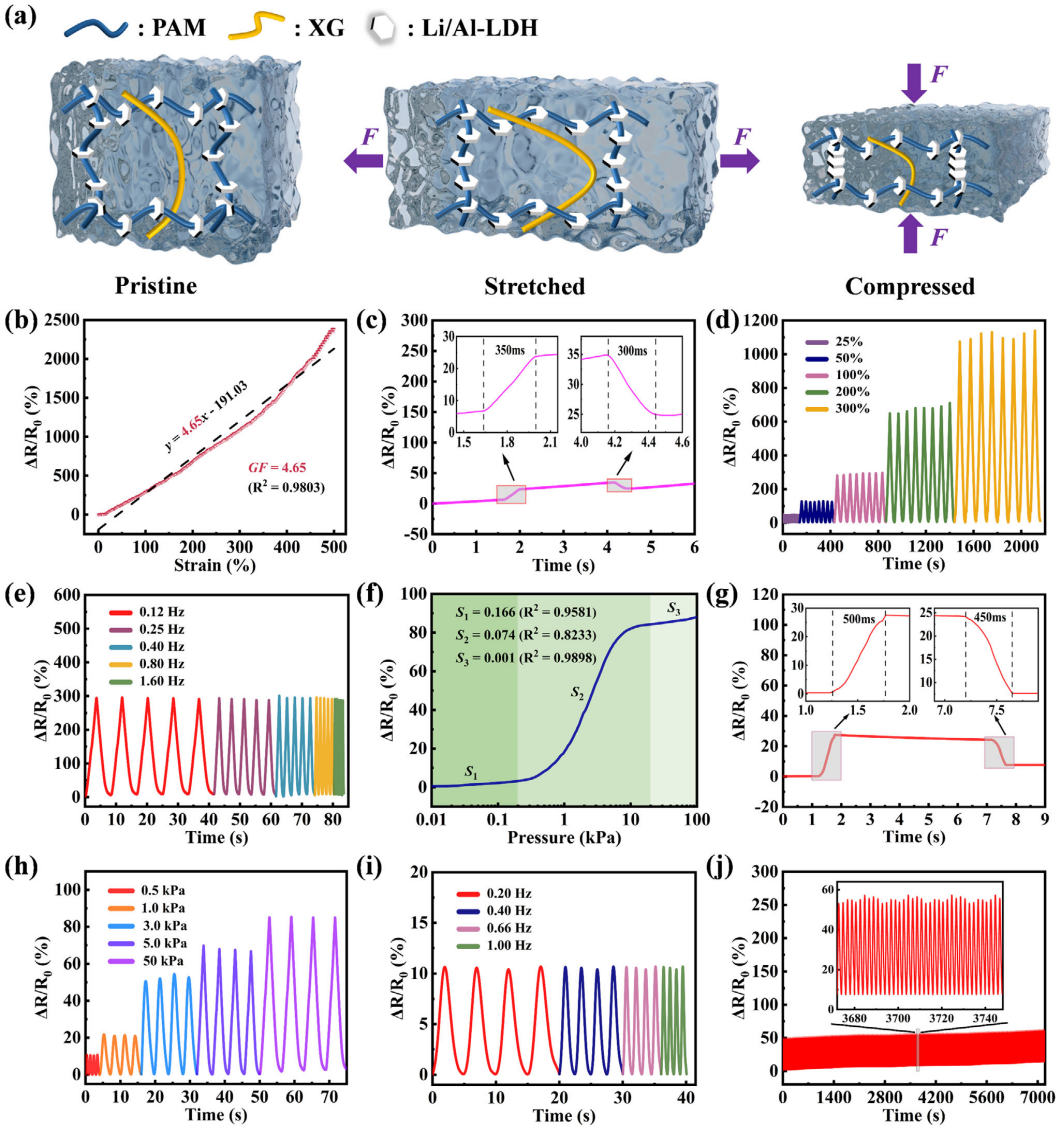
Electromechanical properties of PXL hydrogels. a) The sensing principle of PXL hydrogel under the pristine, stretched, and compressed states. b) The *GF* of PXL_13.33 wt.%_ as a tensile strain sensor (in the strain range of 0–500%, @60 mm min^−1^). c) The tensile response time and relaxation time of the PXL_13.33 wt.%_ sensor (10% strain, @300 mm min^−1^). d) The relative resistance change of the PXL_13.33 wt.%_ strain sensor under different tensile strains (@60 mm min^−1^). e) The relative resistivity change of the PXL_13.33 wt.%_ sensor under 100% tensile strain at different frequencies (0.12–1.6 Hz). f) The sensitivity of the PXL_13.33 wt.%_ sensor under different pressures (0.01–100 kPa, @40 mm min^−1^). g) The compressive response time and relaxation time of the PXL_13.33 wt.%_ sensor (5% strain, @100 mm min^−1^). h) The relative resistivity change of the PXL_13.33 wt.%_ sensor under different pressures (0.5–50 kPa, @80 mm min^−1^). i) The relative resistance change of the PXL_13.33 wt.%_ sensor under a fixed pressure of 0.5 kPa at low frequency (0.2–1.0 Hz). j) PXL_13.33 wt.%_ pressure sensor with 3600 compressive loading‐unloading cycles (3 kPa, 0.5 Hz).

The introduction of Li/Al‐LDH into the hydrogel network reshapes the internal structure, forming a 3D conductive framework that enhances both mechanical properties and strain‐sensing performance.^[^ ^71^ ^]^ When the hydrogel is subjected to external stress, the conductive network undergoes structural changes, leading to resistance variations and enabling strain‐sensing (**Figure** **4**a). An experimental apparatus comprising a digital multimeter, a computer, and a tensile testing machine (Figure S11, Supporting Information) was engineered to characterize the electromechanical properties of PXL. Figure 4b shows the change of the relative resistance of the PXL_13.33 wt.%_ hydrogel during the stretching. The PXL_13.33 wt.%_ nano‐conductive hydrogel shows a good tensile strain response, and the sensitivity reaches *GF* = 4.65 (in the strain range of 0–500%, @60 mm min^−1^). This high sensitivity is attributed to the uniform dispersion of Li/Al‐LDH nanosheets within the PAM matrix, which form strong interfacial bonds and respond effectively to matrix deformation during stretching. To evaluate the repeatability and hysteresis of the PXL sensor, a continuous loading‐unloading cycle test was conducted within a strain range of 500%. As shown in Figure S12a (Supporting Information), the *ΔR/R*_0_ curve exhibits an extremely small hysteresis, with an average hysteresis rate of only 1.45%. Moreover, it shows good coincidence in ten consecutive cycles, which indicates that the sensor has excellent reliability in dynamic sensing applications. In addition, to quantify the resolution and long‐term stability of the PXL sensor, the baseline resistance was recorded for 1 h under the zero‐strain state, and the Allan deviation method was used for analysis. It can be seen from the Allan deviation noise plot (Figure S12b, Supporting Information) that when the averaging time is 7 s, the minimum deviation reaches 0.03%, which represents the optimal resolution of the sensor. Furthermore, the curve remains flat for a relatively long time, indicating excellent long‐term stability and almost no drift—this is particularly important for applications requiring long‐term monitoring.

The tensile response and relaxation times of the PXL_13.33 wt.%_ hydrogel are 350 and 300 ms, respectively (Figure 4c), indicating its rapid response to the stretching. Figure 4d shows the strain‐sensing properties of PXL_13.33 wt.%_ under different strains (25%–300%) during tensile testing, indicating that this material exhibits stable sensing behavior under varying strain conditions.^[^ ^72^ ^]^ Furthermore, the relative resistance change remains stable across different frequencies (0.12–1.6 Hz) when a fixed strain of 100% is applied (Figure 4e), confirming the material's repeatability^[^ ^73^ ^]^ and suitability for real‐time monitoring applications.^[^ ^74^ ^]^

#### 2.4.2 Compressive Electromechanical Properties of PXL Hydrogel

Subsequently, the performance of PXL_13.33 wt.%_ conductive hydrogel in the compressive strain‐sensing was further studied. As shown in Figure 4f, the hydrogel exhibits a relative impedance change of up to 85% within the pressure range of 0.01–100 kPa, with distinct sensitivity levels in three sub‐ranges: 0.01–0.2 kPa (*S*_1_ = 0.166 kPa^−1^), 0.2–20 kPa (*S*_2_ = 0.074 kPa^−1^), and 20–100 kPa (*S*_3_ = 0.001 kPa^−1^) at a strain rate of 40 mm min^−1^. It is worth noting that within the range of 0.2–20 kPa, the sensor not only maintains a wider working range but also possesses a high sensitivity of up to 0.074 kPa^−1^, which shows enormous potential in applications such as flexible wearables.

In terms of response characteristics, the compressive response time and relaxation time of this hydrogel are 500 and 450 ms, respectively (Figure 4g), indicating that it also has a rapid response in the compressive strain‐sensing. Cyclic compression tests at 80 mm min^−1^ within 0.5–50 kPa (Figure 4h) further confirm its stability and reliability in pressure sensing applications.^[^ ^75^ ^]^ In addition, the frequency‐independent sensing behavior of the hydrogel under 0.5 kPa pressure (Figure 4i; Figure S13, Supporting Information) ensures accurate and reliable signal output across low‐ and high‐frequency ranges (0.2–1.0 and 1.3–2.0 Hz), making it suitable for real‐time human motion detection.^[^ ^18^ ^]^

Long‐term stability was assessed through 3600 compressive and 3000 tensile loading–unloading cycles (Figure 4j; Figure S14, Supporting Information), revealing negligible signal drift. The mechanical and electromechanical properties of the PXL_13.33 wt.%_ hydrogel placed at 25 °C for 7 days were tested. By comparing the data obtained 7 days ago, it can be seen that the reduction in the tensile strength of the hydrogel is negligible, and it still maintains a tensile sensitivity of 4.25 and can remain stable in the tensile sensing property tests under different deformations (Figure S15a–c, Supporting Information). This proves that the PXL_13.33 wt.%_ hydrogel can be used stably even if placed uncovered at room temperature for more than a week.

### 2.5 Demonstration of Multifunctional Sensors Based on PXL Hydrogel

#### 2.5.1 Voice Recognition

**Figure 5:**
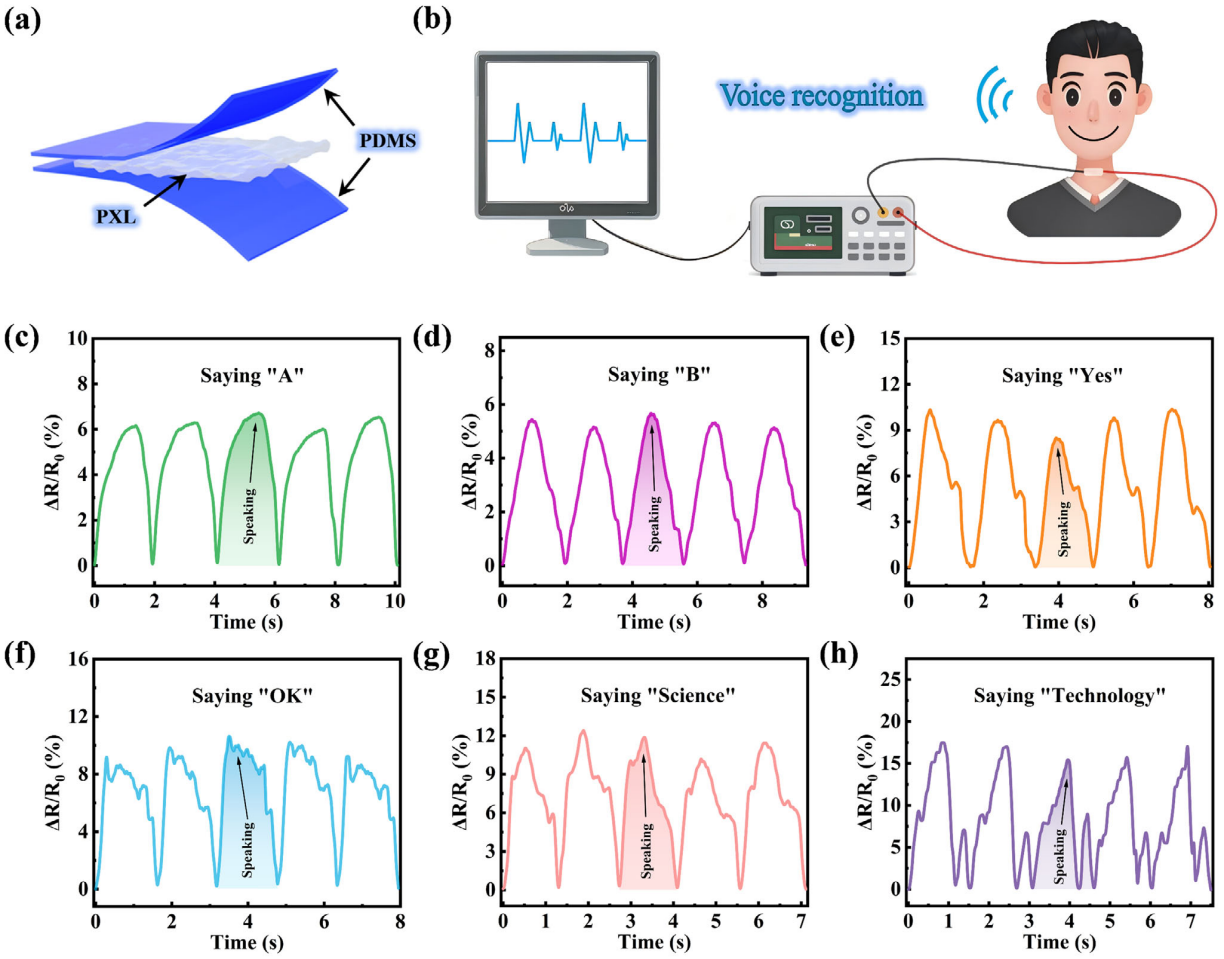
The wearable sensor based on PXL is used for human speech recognition. a) Schematic diagram of the structure of the PXL wearable sensor. b) Schematic diagram of the connection of the PXL hydrogel for speech recognition. The relative resistance changes of the hydrogel when the wearer says simple single letters c) “A” and d) “B,” short syllables e) “Yes” and f) “OK,” and complex syllables g) “Science” and h) “Technology.”.

To further verify the application feasibility of PXL hydrogel in the sensing field, it was further assembled and encapsulated into a wearable sensor. The sensor adopts a sandwich structure with two PDMS outer layers and a central PXL hydrogel layer, enabling electrical connectivity and real‐time signal transmission (**Figure** **5**a). The PDMS encapsulation ensures synchronized deformation with the monitored area, protects the hydrogel from environmental interference, and reduces water loss, enhancing long‐term stability under complex conditions. Before human behavior testing, the PXL_13.33 wt.%_ hydrogel was tested for biocompatibility by attaching it to human skin for three days. No allergic reactions such as itching or redness were observed, confirming its biocompatibility (Figure S16, Supporting Information). The excellent biocompatibility ensures the safe application of this hydrogel in the field of flexible wearable electronics.^[^ ^76^ ^]^

As shown in Figure 5b, the encapsulated PXL hydrogel sensor is attached to the vocal cords of volunteers for real‐time behavior monitoring and sensing. When volunteers speak, vocal cord vibrations act on the hydrogel, converting biological signals into analog signals that are collected by a multimeter, transmitted to a computer, and finally output as digital signals via a LABVIEW program. Figure 5c–h shows the electrical signals generated from single letters to short syllables and then to complex syllables. It is clearly observable that the waveform of each pronunciation exhibits uniqueness. Following five repeated trials, the electrical signal waveforms demonstrated remarkable consistency, thereby validating the hydrogel's applicability for monitoring of sound waves.

#### 2.5.2 Motion Recognition

**Figure 6:**
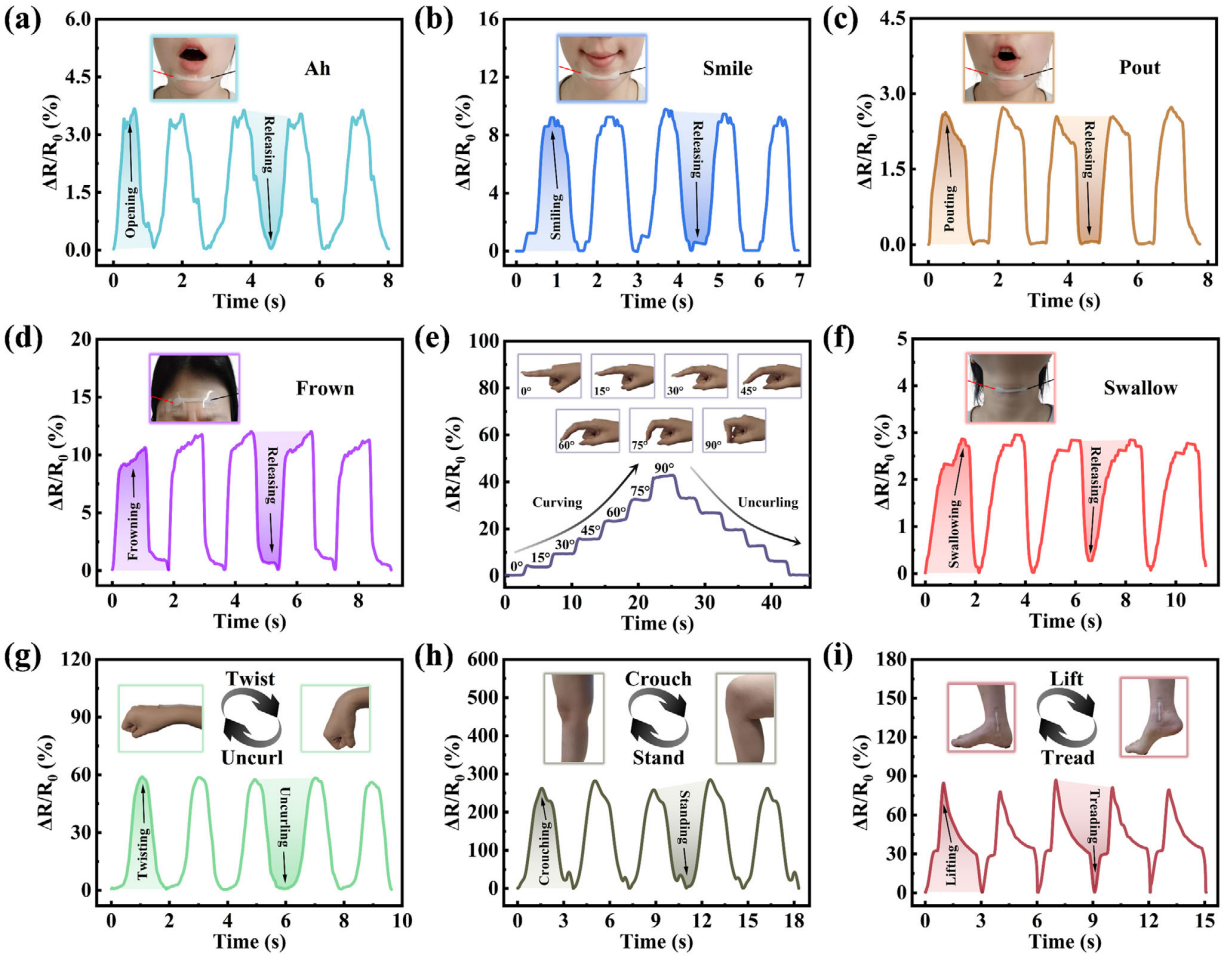
The wearable sensor based on PXL is used for human behavior sensing. The electrical wave diagrams when volunteers make these subtle facial expressions of a) “Ah,” b) “Smile,” c) “Pout,” and d) “Frown.” e) The relative resistance change of the PXL_13.33 wt.%_ sensor attached to the volunteer's finger when the curving angle increases from 0° to 90° and then recovers. Signal monitoring of f) swallowing, g) wrist and h) knee bending, and i) lifting the foot for these large‐scale human movements.

As shown in **Figure** **6**a–d, the sensor can detect changes in facial expressions, such as “ah,” “smile,” “pout” and “frown,” by capturing distinct electrical signals generated during these actions.^[^ ^77^ ^]^ When fixed on the index finger, the sensor records electrophysiological signals during the full range of motion from 0° to 90° and back,^[^ ^78^ ^]^ exhibiting a step‐like response that clearly demonstrates the status of each bending action (Figure 6e). The resistance changes induced by deformation are consistent at each angle and return to the initial value, confirming the sensor's stability and sensitivity.^[^ ^79^ ^]^

Furthermore, by being worn on the human body (Figure S17, Supporting Information), the sensor based on PXL_13.33 wt.%_ can also detect the electrical signals generated by small‐amplitude movements such as neck swallowing (Figure 6f), as well as the electrical signals generated by large‐amplitude movements such as wrist twisting (Figure 6g), knee deformation during crouching (Figure 6h), and foot lifting (Figure 6i), demonstrating its high sensitivity and fatigue resistance.^[^ ^80^ ^]^ Long‐term stability was tested by placing the sensor at room temperature for 7 days, with no significant degradation in sensing performance (Figure S18a–c, Supporting Information). This proves that the PXL_13.33 wt.%_ can be used stably even in a relatively dry environment. Therefore, this hydrogel has promising potential applications in the fields of environmental detection, medicine, and human health monitoring.^[^ ^81^ ^]^

### 2.6 Demonstration of Practical Applications Based on PXL Hydrogel

#### 2.6.1 8 × 8 Array Sensor

**Figure 7:**
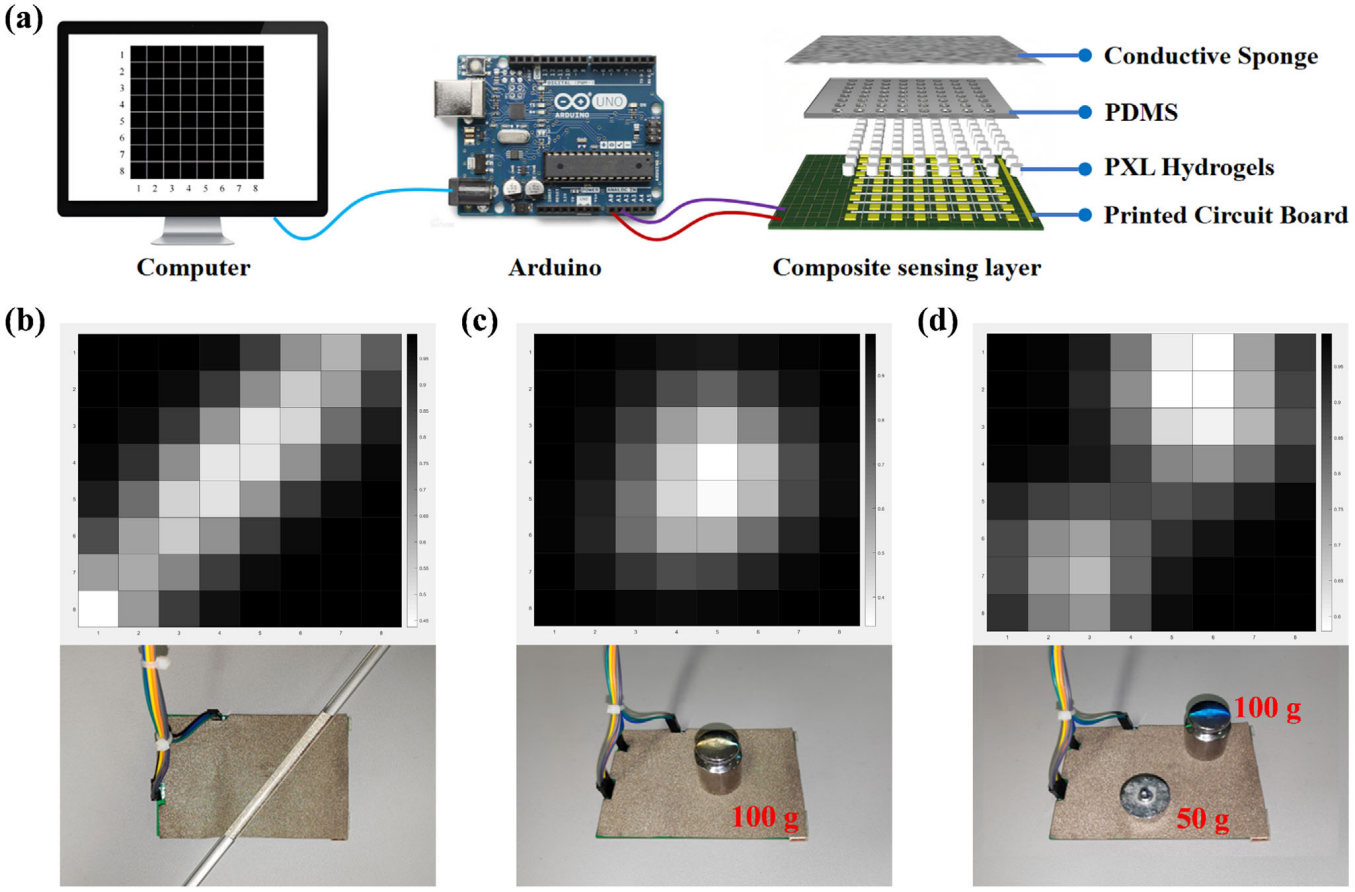
Array sensing device based on PXL hydrogel for pressure visualization. a) Structural composition and physical connection diagram of the array sensor. Visual color block displays corresponding to b) glass rod, c) weight, and d) weights of different masses.

As shown in **Figure** **7**a, the hydrogel is further assembled and encapsulated. Sixty‐four small columnar hydrogels are placed on the designed circuit board, and PDMS is used to fix the position of the hydrogels to reduce the influence of other factors on the hydrogels. PDMS is a flexible polymer chain with high viscoelasticity and is used for synchronous stretching and deformation with the monitoring part. Finally, it is encapsulated with conductive sponge to form a closed circuit to construct an 8 × 8 hydrogel array sensor. The schematic diagram of the array sensor is shown in Figure S19 (Supporting Information). The sensor operates by measuring voltage changes across a fixed resistor via series voltage division, which is then mapped to grayscale color blocks in a MATLAB program for real‐time pressure visualization.

Experiments demonstrate that the sensor can accurately detect and visualize the shape and weight (Figure 7b,c) of objects placed on it, with each hydrogel cell functioning independently without cross‐interference. When multiple objects or weights were applied simultaneously (Figure 7d), the color blocks displayed distinct gray levels corresponding to the pressure distribution, confirming the sensor's reliability and scalability for complex sensing tasks. Video S2 (Supporting Information) showcases the detailed implementation process of this experiment. The successful practice of this experimental design fully confirms the enormous application potential of PXL hydrogel in the field of flexible wearable devices.^[^ ^82^ ^]^

#### 2.6.2 Three‐Point Plantar Pressure‐Sensing Smart Insole

**Figure 8:**
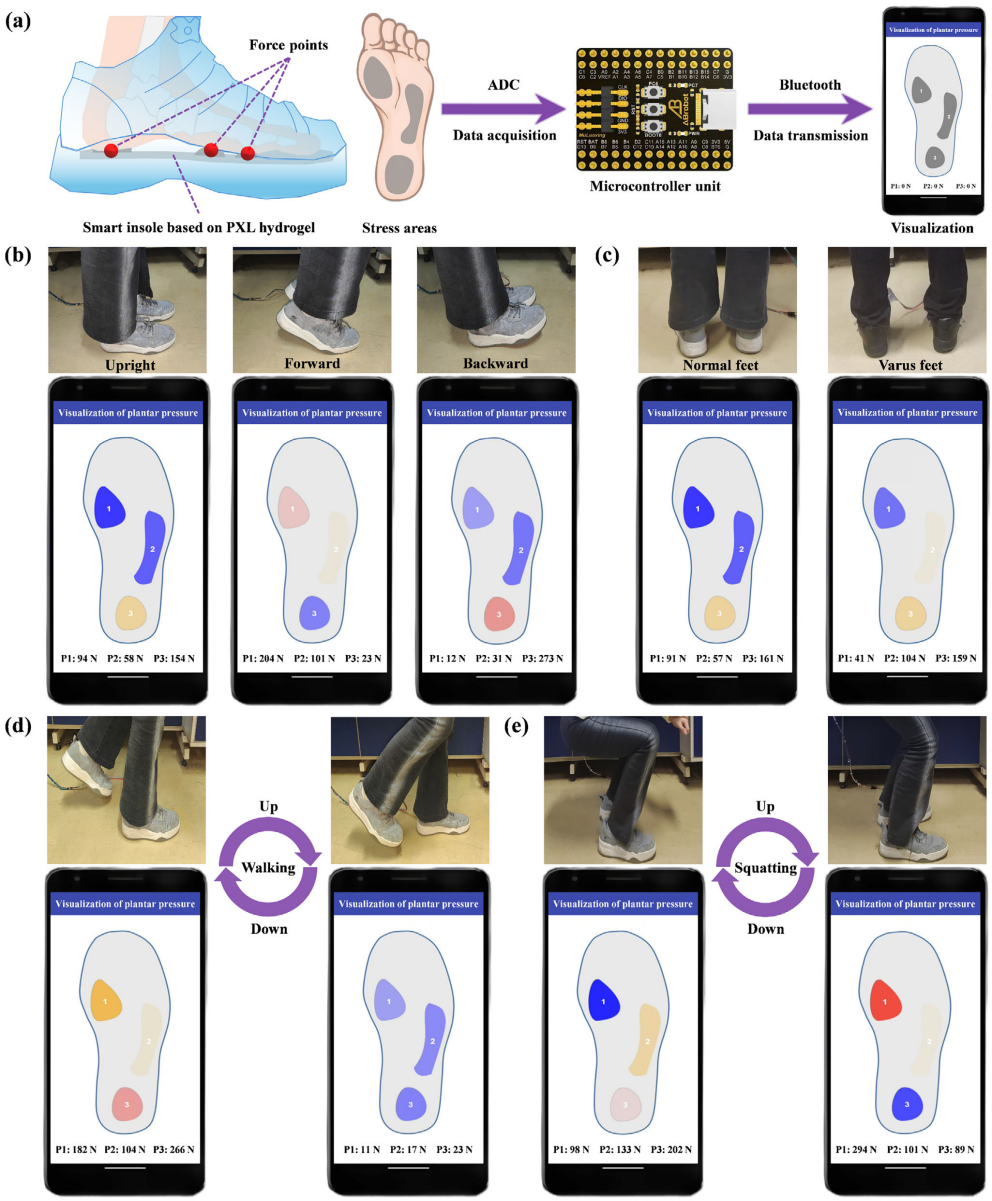
The plantar pressure monitoring system is based on column‐shaped PXL hydrogel pressure sensors. a) The main framework of the plantar pressure monitoring system. Real‐time visualization of plantar pressure distribution corresponding to b) different standing postures, c) different foot types, d) lowering and lifting the foot during walking, and e) squatting down and standing up during the squatting process.

To achieve real‐time monitoring of plantar pressure amplitude and spatial distribution, a plantar pressure monitoring system is developed based on columnar PXL hydrogel pressure sensors. As shown in **Figure** **8**a, the system consists of a smart insole for pressure detection, an analog‐to‐digital converter (ADC) for data acquisition, a microcontroller for data processing, a Bluetooth module for wireless transmission, and a mini‐program interface for visualizing pressure distribution. The system enables real‐time monitoring of pressure distribution across the three primary regions of the foot under various postures. These three positions are the first metatarsal head (located at the base of the big toe), the fifth metatarsal head (located at the base of the little toe), and the heel—they are the main weight‐bearing areas of the plantar region. By monitoring the data from these points, the mechanical state of the entire foot can be roughly inferred or estimated. On the display interface, pressure ranges (0–100, 101–200, and 201–300 N) are mapped to colors (blue, yellow, red), with darker shades indicating higher pressure. This color‐based visualization mimics the visual perception of pressure changes.

First, the system's ability to monitor plantar pressure in static conditions is tested. As shown in Figure 8b, the pressure distribution was tested under three postures: upright, forward‐leaning, and backward‐leaning. In the upright posture, pressure is evenly distributed, with a slight concentration on the heel, resulting in a light yellow signal (154 N) for the heel and blue signals (94 and 58 N) for the inner and outer forefoot, respectively. When leaning forward, pressure shifts to the forefoot, with increased load on the inner side, causing the inner forefoot sensor to display light red (204 N), the outer forefoot to show light yellow (101 N), and the heel to remain light blue (23 N). In the backward‐leaning posture, pressure is mainly concentrated on the heel, resulting in a darker red signal (273 N) for the heel and blue signals (12 and 31 N) for the inner and outer forefoot, respectively. Figure 8c further demonstrated the system's ability to distinguish between normal and varus foot types. The varus foot exhibited greater pressure on the lateral side, causing the corresponding sensor to display a lighter yellow (104 N). These results are consistent with the law of human plantar pressure distribution, confirming the accuracy of the system in capturing and visualizing plantar pressure distribution under different postures and foot types, highlighting its potential for applications in gait analysis, orthopedic diagnostics, and personalized footwear design.

The plantar pressure monitoring system is further validated for its ability to track real‐time changes in plantar pressure during dynamic human movements. As shown in Figure 8d, the system successfully captured the pressure distribution during the lifting and lowering of the foot during walking. When the foot is lifted, all pressure‐sensor channels display blue signals (11, 17, and 23 N), indicating basically no pressure. Upon reapplication of the foot, the first two sensor channels shift to yellow (182 and 104 N), and the last channel turns red (266 N), reflecting the localized pressure increase on the forefoot and heel. This rapid response confirms the system's real‐time monitoring capability. In Figure 8e, the system was tested during squatting movements. During the squatting phase, pressure on the heel and the outer sole increases significantly, causing the corresponding sensor channels to display red (202 N) and yellow (133 N), respectively. During the standing‐up phase, the forefoot becomes the primary load‐bearing area, with the inner side of the sole experiencing the highest pressure, resulting in a deep red signal (294 N) in the inner forefoot sensor. The demonstration effects of some actions in this experiment are shown in Video S3 (Supporting Information). These results demonstrate the system's ability to accurately reflect the spatial and temporal distribution of plantar pressure during dynamic activities.

The system's real‐time tracking and visualization of plantar pressure changes enable the prediction of exercise‐induced fatigue and the correction of improper force‐generating postures during physical activity. This capability supports its application in diverse fields, including human posture analysis, rehabilitation, sports training, and health monitoring.

## 3 Conclusion 

In summary, by in situ polymerization of PAM between Li/Al‐LDH nanosheets and forming a semi‐interpenetrating network with XG, a multifunctional nano‐conductive hydrogel PXL with high conductivity, high elongation, and high sensitivity has been successfully prepared, which shows promising applications in the field of strain‐sensing. Using a freshly prepared Li/Al‐LDH solution with the Tyndall effect enables in situ polymerization of acrylamide between Li/Al‐LDH nanosheets, ensuring the uniform distribution of Li/Al‐LDH nanosheets in the hydrogel network. Molecular dynamics simulations demonstrate that the incorporation of Li/Al‐LDH nanosheets endows the hydrogel with exceptional mechanical properties. The abundant metal cations in Li/Al‐LDH engage in coordination or complexation interactions with functional groups on both PAM and XG, enabling the hydrogel to simultaneously achieve superior sensing performance and high mechanical strength. Therefore, the PXL hydrogel shows a tensile strength at break as high as 208 kPa and a strain at break as high as 2350%, as well as an electrical conductivity as high as 2.08 S m^−1^ and a high tensile sensitivity as high as 4.65. PXL can also sense pressure changes and has a high‐pressure sensitivity of 0.074 kPa^−1^ within the pressure range of 0.2–20 kPa, as well as long‐term stability for more than 3600 consecutive compressive cycles. PXL can monitor human movements from acoustic vibrations to complex deformations and is expected to provide effective help for special groups such as people with speech impairments. In addition, with PXL hydrogel as the core sensing material, practical applications such as the 8 × 8 array sensor and the three‐point plantar pressure‐sensing smart insole have been constructed, demonstrating the excellent sensing performance of the hydrogel and indicating that the nano‐conductive hydrogel has great application potential in wearable and flexible devices.

## Conflict of Interest

The authors declare no conflict of interest.

## Participants and Ethics Statement

It was confirmed that informed written consent from all participants was obtained prior to the experiments with sensors and wearable technologies. The strain sensor was encapsulated with PDMS and did not directly contact human skin. Additionally, only resistance changes of the hydrogel sensor were obtained during the test and no information regarding the human beings was obtained. Therefore, the approval from a national or institutional ethics committee was not required in this case.

## Supporting information



Supporting Information

Supporting Information

Supplementary Video

Supplementary Video

Supplementary Video

## Data Availability

The data that support the findings of this study are available from the corresponding author upon reasonable request.
