# Supplementary material for: Li/Al‐LDH Reinforced Polyacrylamide/Xanthan Gum Semi‐Interpenetrating Network Nano‐Conductive Hydrogels for Stress Sensing and Wearable Device Applications
Source: Adv Sci (Weinh). 2025 Sep 30;12(47):e11903. doi: 10.1002/advs.202511903 (PMC12713048; doi:10.1002/advs.202511903)
Supplement: Supplementary file 1 — Supporting Information [file ADVS-12-e11903-s003.docx]

Supporting Information

**Li/Al-LDH Reinforced Polyacrylamide/Xanthan Gum Semi-Interpenetrating Network Nano-Conductive Hydrogels for Stress Sensing and Wearable Device Applications**

*Zhiwei Hu, Tuo Li, Yong Zheng, Shengxi Chen, Tong Wan, Hamdy Khamees Thabet, Zeinhom M. El-Bahy, Dalal A. Alshammari,* *Hanhui Lei, Liqiang Chu, Yunlong Sun, Yaohui Guo, Yizhou Yang, Terence Xiaoteng Liu*, Dapeng Cui, Zhanhu Guo, Huige Wei****

Zhiwei Hu, Tuo Li, Yong Zheng, Shengxi Chen, Tong Wan, Liqiang Chu, Huige Wei

State Key Laboratory of Bio-based Fiber Materials, Tianjin Key Laboratory of Brine Chemical Engineering and Resource Eco-Utilization, College of Chemical Engineering and Materials Science, Tianjin University of Science and Technology, Tianjin 300457, China

Email: [huigewei@tust.edu.cn](mailto:huigewei@tust.edu.cn)

Yunlong Sun, Dapeng Cui

College of Light Industry Science and Engineering, Tianjin University of Science and Technology, Tianjin, 300457, China

Yaohui Guo, Yizhou Yang

College of Electronic Information and Automation, Tianjin University of Science and Technology, Tianjin, 300457, China

Hanhui Lei, Terence Xiaoteng Liu

Faculty of Engineering and Environment, Northumbria University, Newcastle Upon Tyne NE1 8ST, UK

Email: [terence.liu@northumbria.ac.uk](mailto:terence.liu@northumbria.ac.uk)

Zhanhu Guo

Department of Mechanical and Construction Engineering, Northumbria University, Newcastle Upon Tyne NE1 8ST, UK

Hamdy Khamees Thabet

Center for Scientific Research and Entrepreneurship, Northern Border University, Arar 73213, Saudi Arabia

Zeinhom M. El-Bahy

Department of Chemistry, Faculty of Science, Al-Azhar University, Nasr City 11884, Cairo, Egypt

Dalal A. Alshammari

Department of Chemistry, College of Science, University of Hafr Al Batin, Hafr Al Batin P.O. Box 39524, Saudi Arabia

Zhiwei Hu, Tuo Li contributed equally to this work


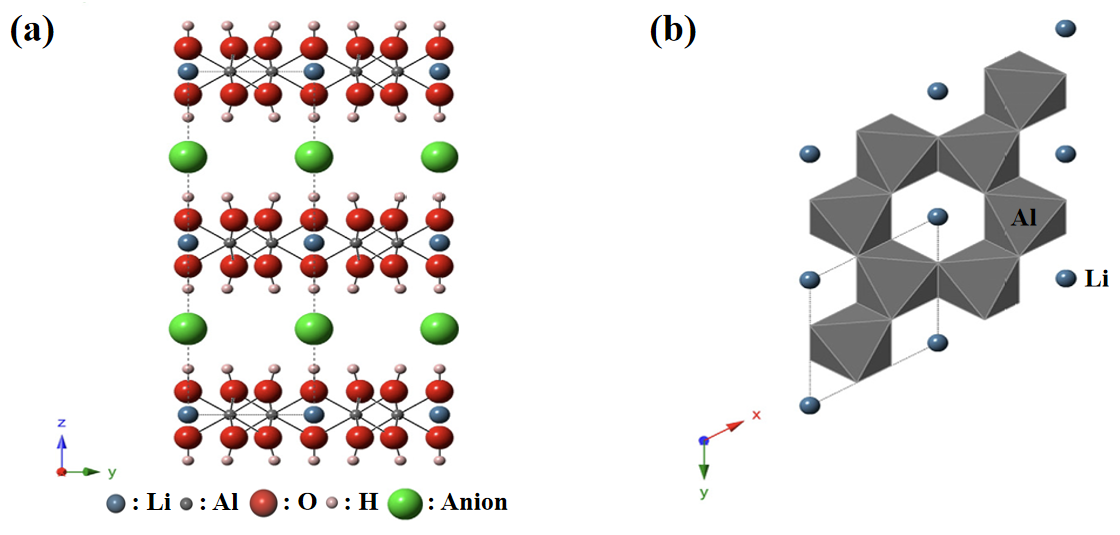


**Figure S1.** Crystal structure of Li/Al-LDH projected along the (a) x axe and (b) z axe, respectively.


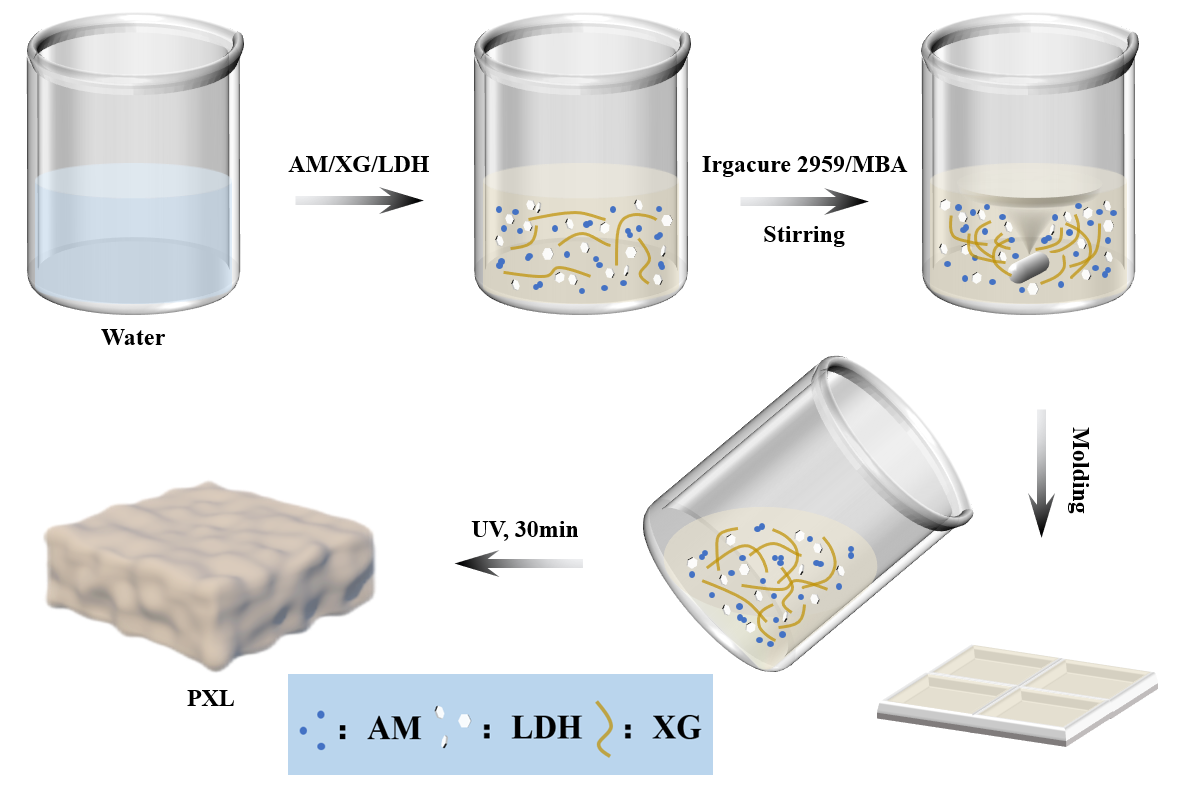


**Figure S2.** Detailed preparation process of PXL hydrogel.


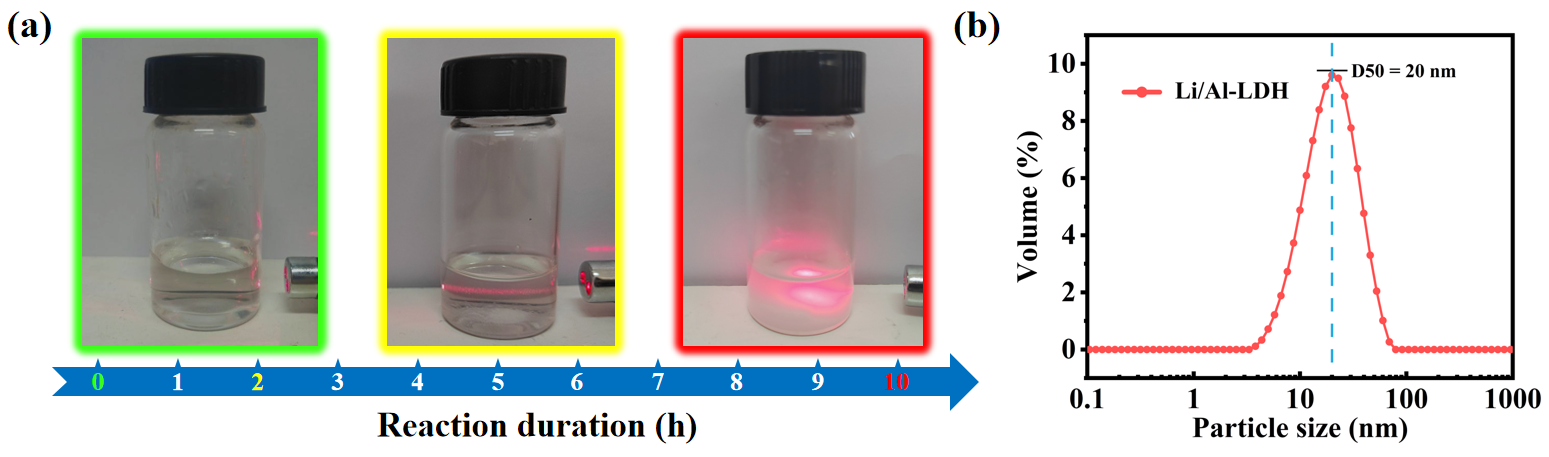


**Figure S3.** (a) The phenomena of Li/Al-LDH solution under the irradiation of a red laser pointer at different reaction durations. (b) The particle size distribution of Li/Al-LDH.


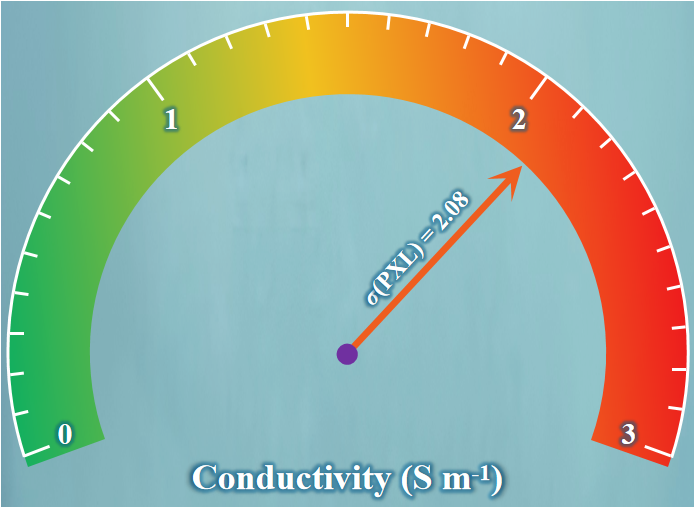


**Figure S4.** The electrical conductivity of PXL at 25 ℃


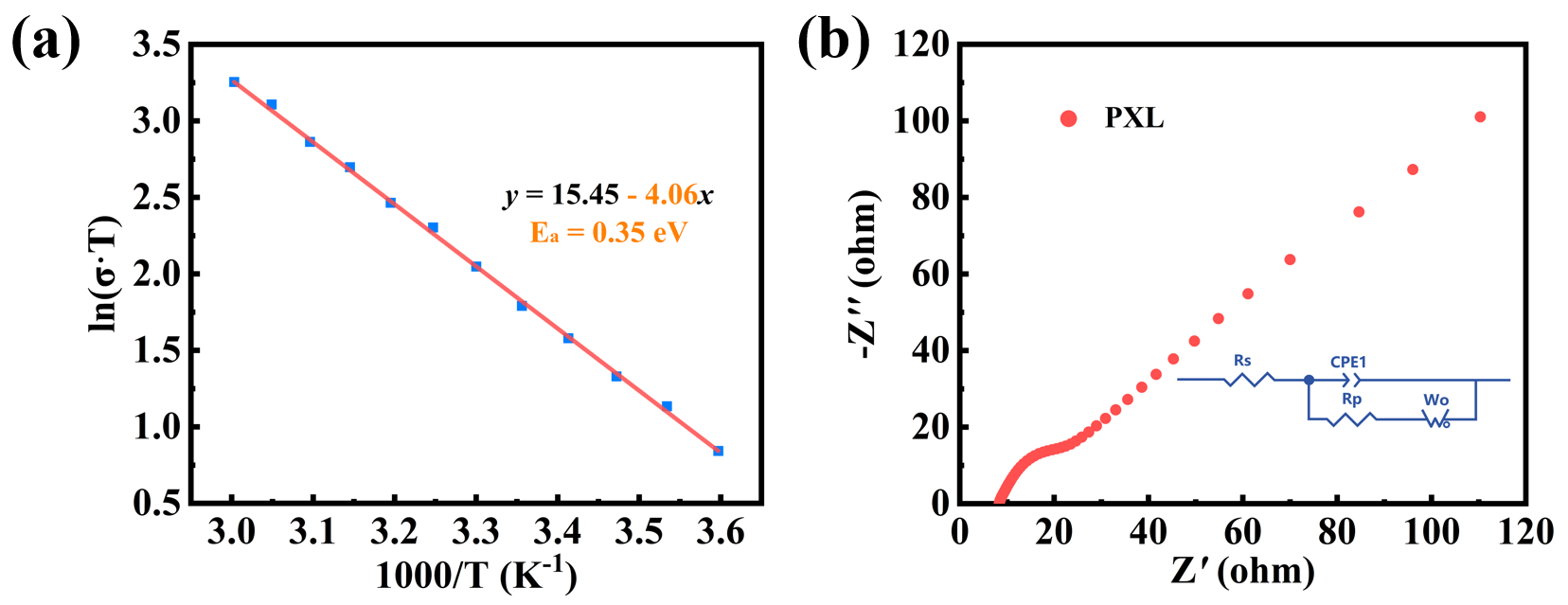


**Figure S5.** (a) Arrhenius plot of PXL hydrogel for variable-temperature conductivity test at 5 - 60 ℃. (b) Nyquist plot of PXL hydrogel at 25 ℃.


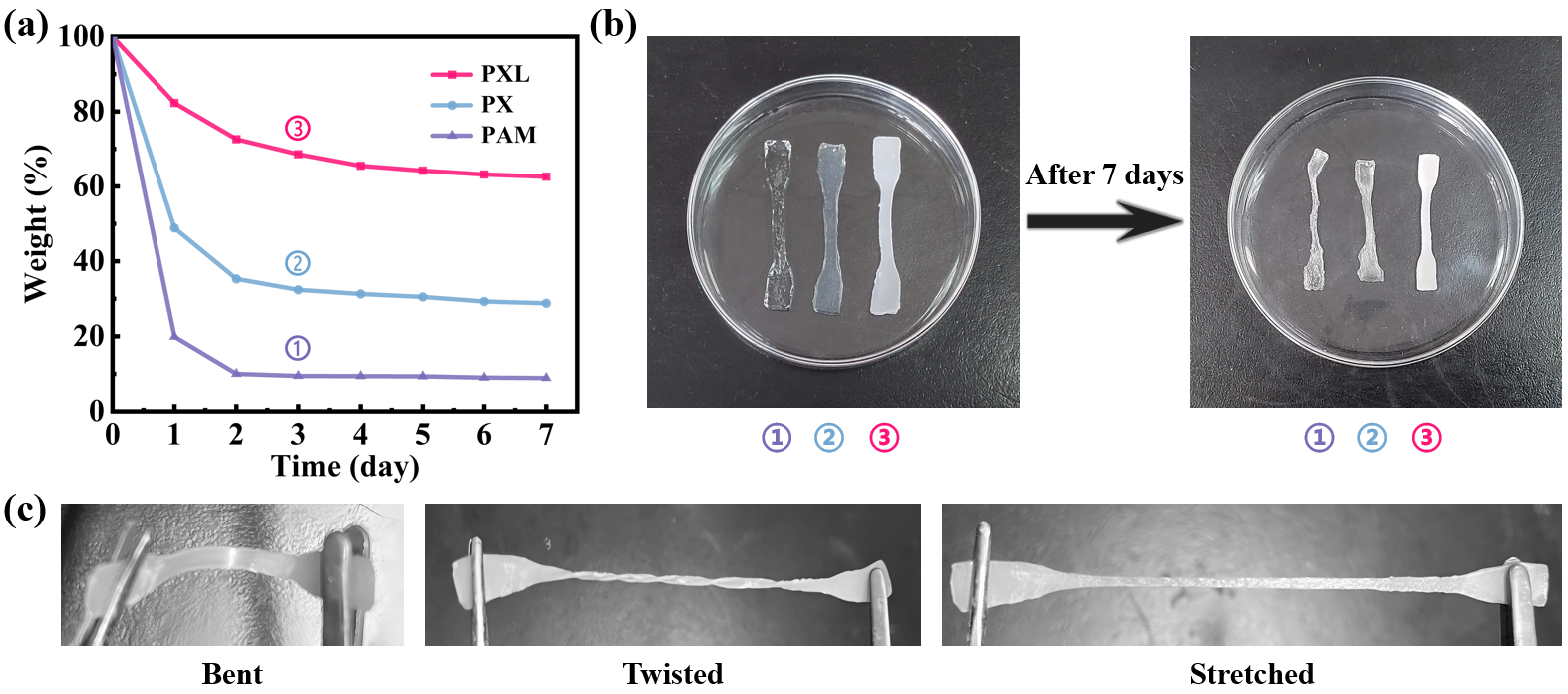


**Figure S6.** (a) Weight changes of PAM, PX and PXL for 7 consecutive days at 25℃. (b) Comparison of digital photographs of PAM, PX and PXL before and after 7 days at 25℃. (c) Digital photographs of the PXL hydrogel after being placed for 7 days undergoing various deformations under the action of external forces.


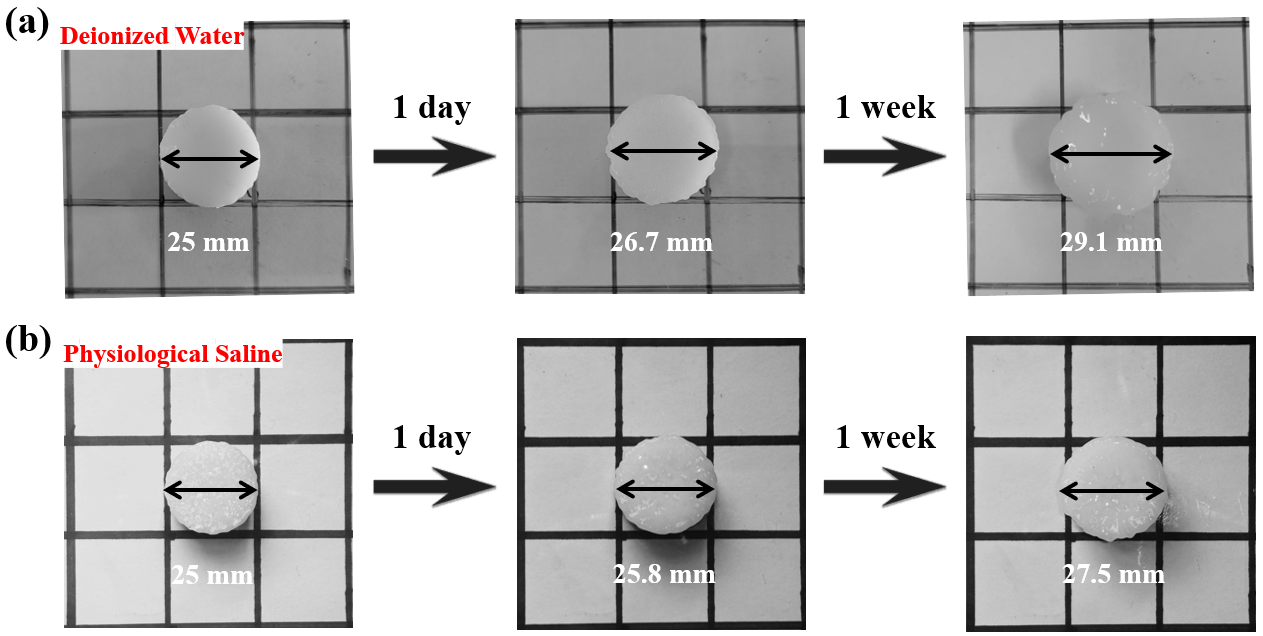


**Figure S7.** The swelling behavior of PXL hydrogel in (a) deionized water and (b) physiological saline

**The molecular dynamics simulation process of PXL**

The content of each component in all the hydrogel systems used for simulation is for the sample size of one unit cell (uc). As shown in Figure S8a, the AC model of the PAM hydrogel was prepared by randomly placing PAM oligomeric chains, MBA monomers, and water molecules together in a cubic simulation cell to obtain the initial hydrogel sample. The sample was initially generated at a low density and then geometrically optimized to ensure that the molecules were fully mixed in the simulation unit before the cross-linking process started. During the cross-linking process, cross-linking bonds were formed in real time between the PAM oligomers and MBA molecules through an in-situ dynamic cross-linking strategy. This dynamic cross-linking method was previously established by Demir and Walsh for epoxy resin systems^[1]^. These newly formed covalent bonds dynamically generated between the PAM oligomers and MBA molecules eventually constructed a three-dimensional polymer network structure. In detail, for the dynamic cross-linking process, the cutoff distance was increased stepwise first, and then the reactive atomic site pairs (located at the chain ends of the PAM oligomers and MBA monomers) were searched for multiple times within the same cutoff distance, and covalent cross-linking bonds were formed between them^[2]^. Finally, the sample was relaxed through a multi-step relaxation procedure to relieve the potential high internal stress generated by the initial formation of new bonds. This process was repeated until the target degree of cross-linking was achieved.

In the simulation of the stress-strain test, the hydrogel sample was subjected to a stepwise applied stress at a constant tensile strain rate in each principal direction^[3]^. Figure S8a also shows representative snapshots of the stretched PAM hydrogel sample under different stresses, indicating that the shape of the simulation unit can change to varying degrees in response to the externally applied stress. For the molecular dynamics simulations of the PX (Figure S8b) and PXL (Figure S8c) hydrogels, except for the differences in the process of establishing the initial hydrogel model, the remaining cross-linking simulations and stress-strain simulations are the same as described above.


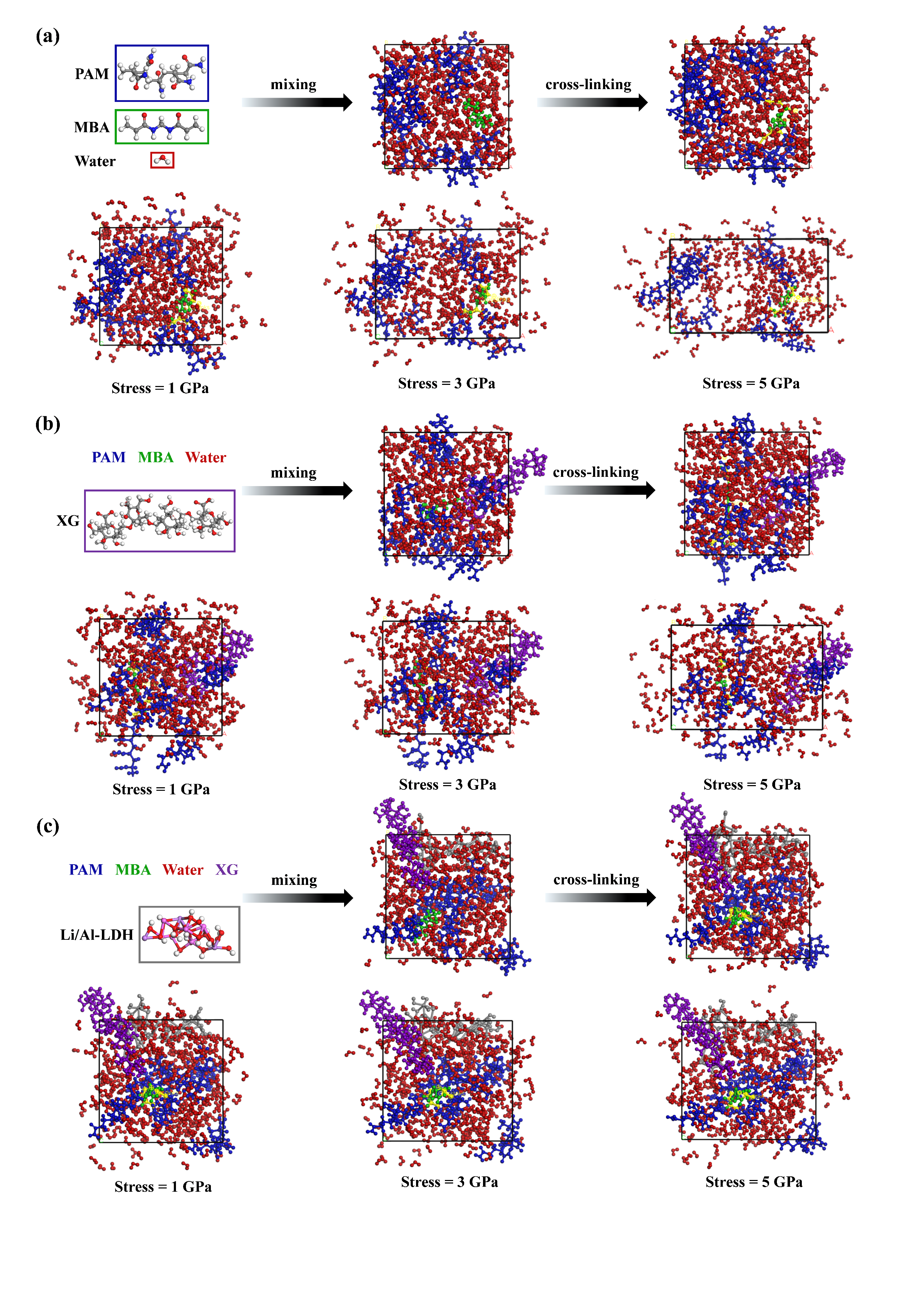


**Figure S8.** (a) The cross-linking simulation process of the PAM hydrogel and the strain states under different stresses. (b) The cross-linking simulation process of the PX hydrogel and the strain states under different stresses. (c) The cross-linking simulation process of the PXL hydrogel and the strain states under different stresses.


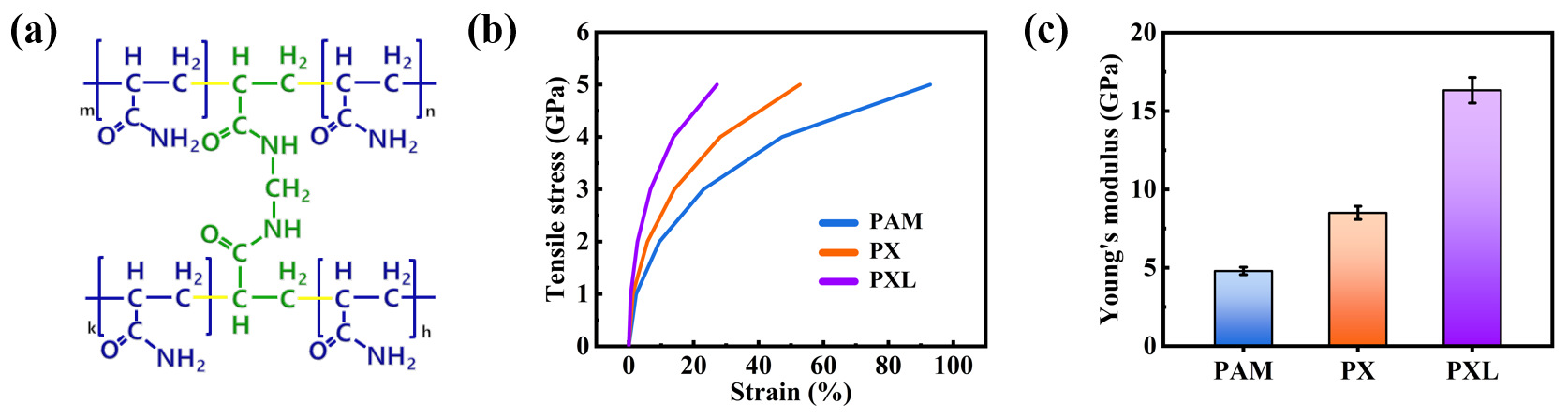
 **Figure S9.** (a) The cross-linking principle of PAM and MBA. (b) The stress-strain curves of PAM, PX and PXL hydrogels under a stress of 5 GPa. (c) The Young's moduli of PAM, PX and PXL hydrogels.


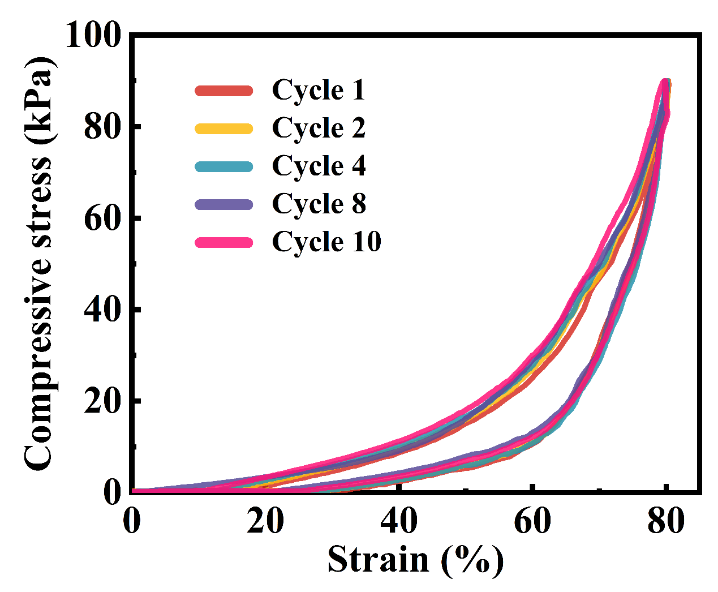


**Figure S10.** Ten continuous compression cycles at 80% strain without resting.


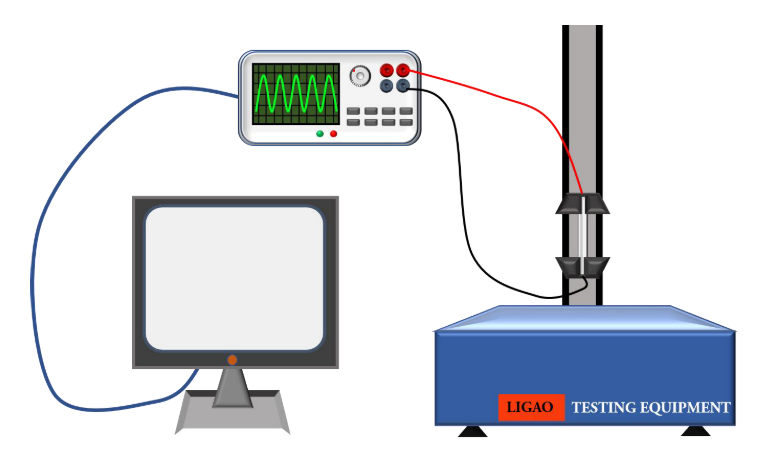


**Figure S11.** The electrical property evaluation device is composed of a tensile machine, a multimeter and a computer.


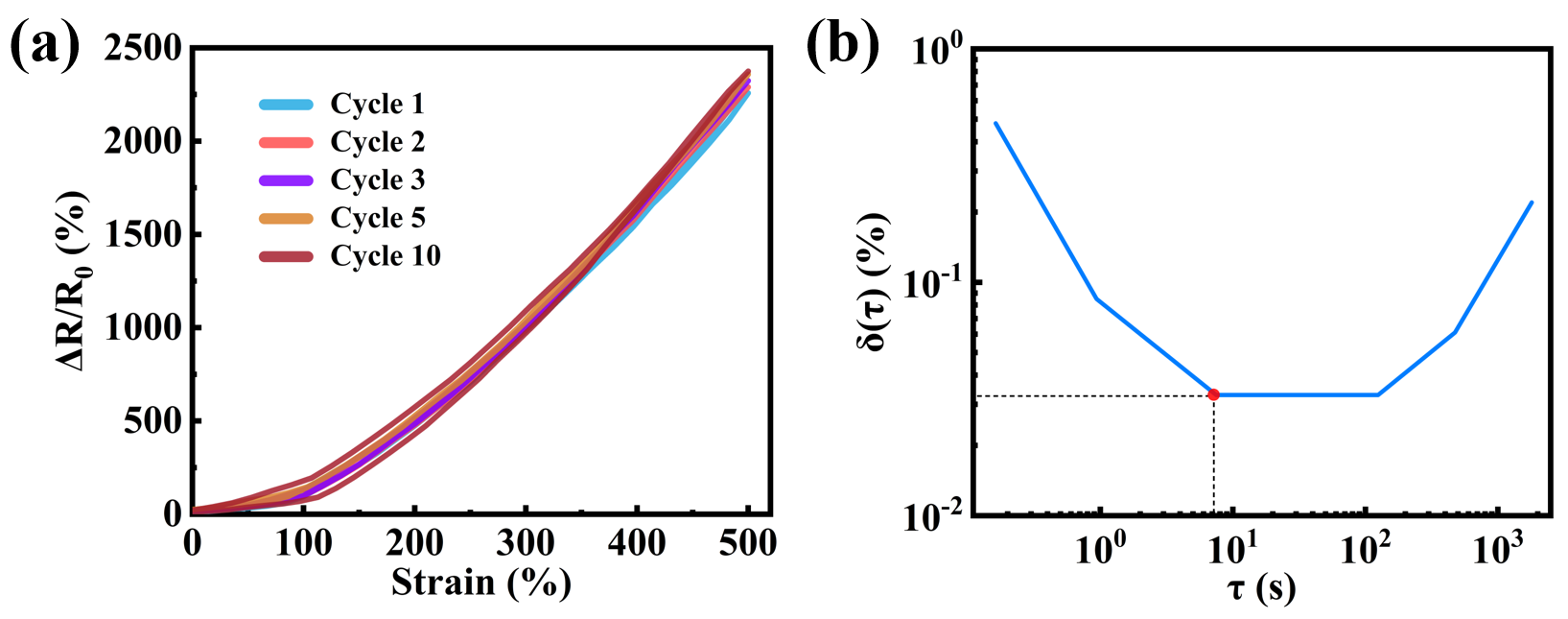


**Figure S12.** (a) The relative resistance change of the PXL_13.33 wt%_ strain sensor under cyclic (loading/unloading) conditions at a 500% tensile strain (@60 mm min^-1^). (b) Allan deviation noise plot of the PXL_13.33 wt%_ strain sensor.


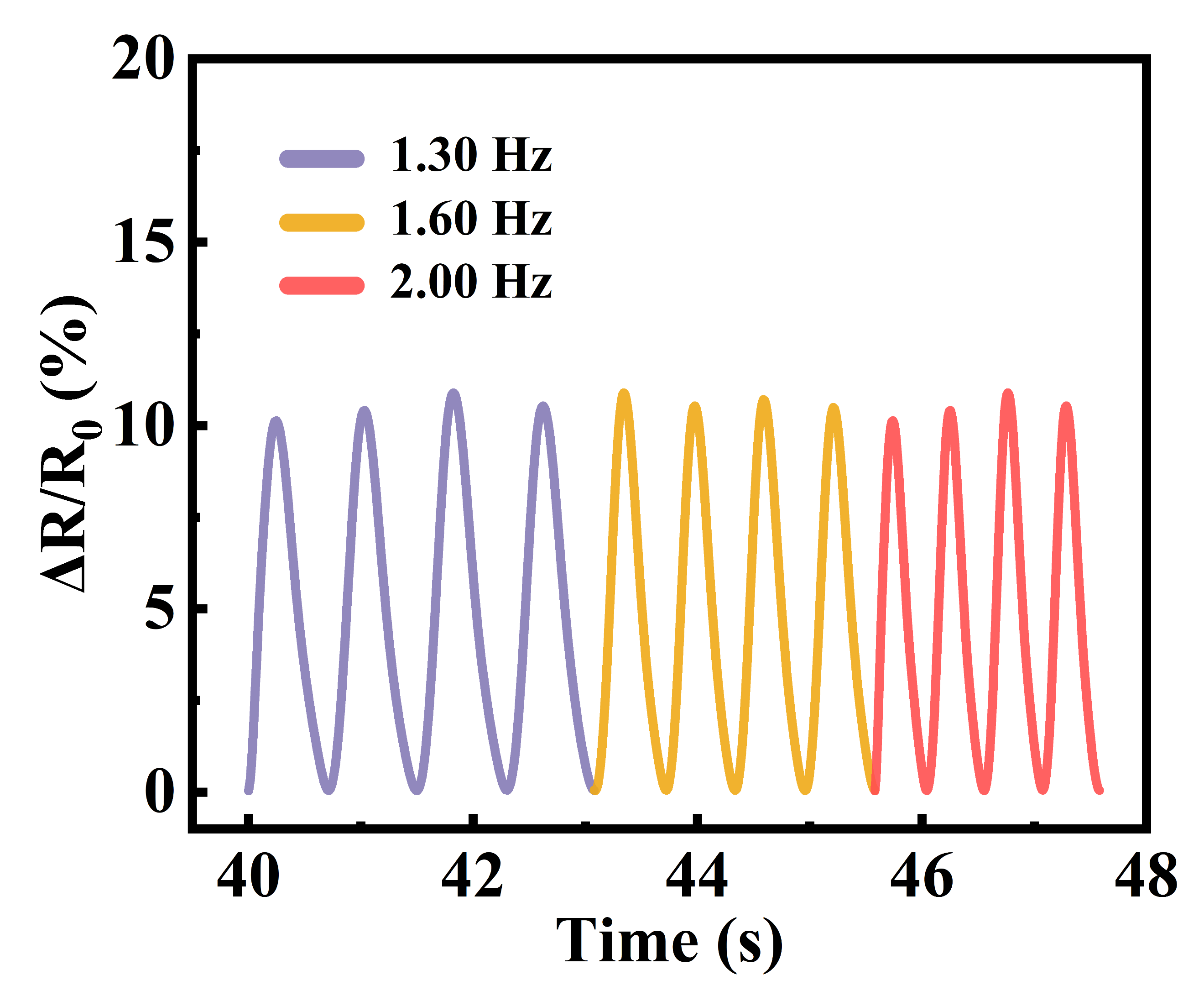


**Figure S13.** The relative resistance change of the PXL_13.33 wt%_ sensor under a fixed pressure of 0.5 kPa at high frequency (1.3 - 2.0 Hz).


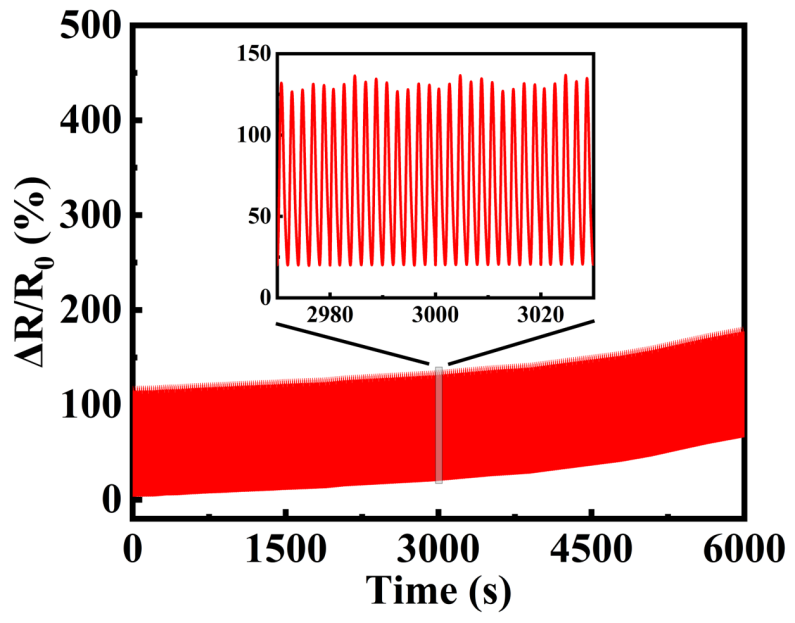


**Figure S14.** PXL_13.33 wt%_ strain sensor with 3000 tensile loading-unloading cycles (50% strain, 0.5 Hz).


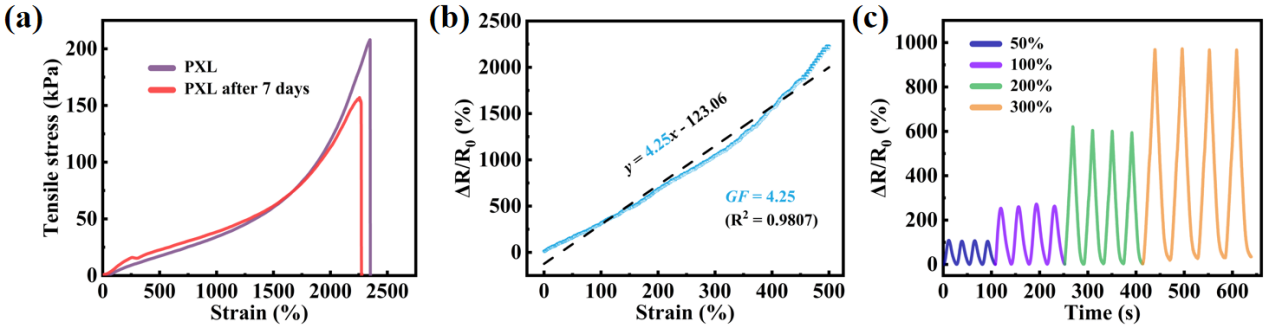


**Figure S15.** (a) Stress-strain curves of PXL hydrogel before and after being placed at room temperature of 25°C for 7 days. (b) *GF* and (c) relative resistance changes at different strains (@60 mm·min^-1^) of PXL hydrogel after being placed at room temperature of 25°C for 7 days.


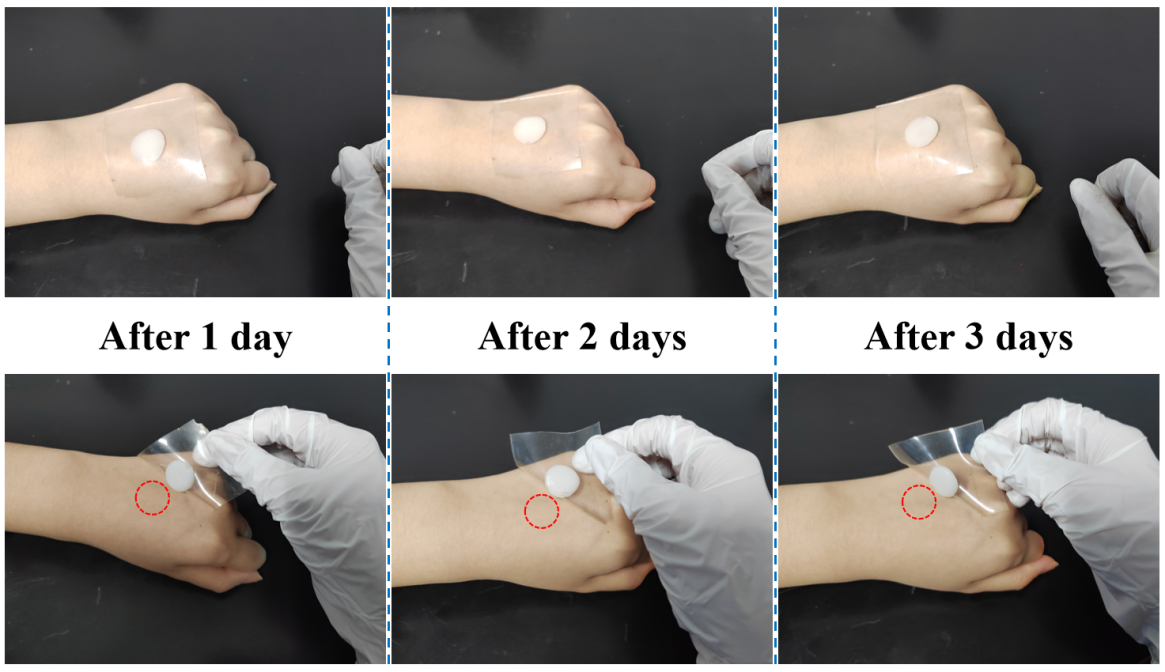


**Figure S16.** Biocompatibility test of PXL hydrogel.


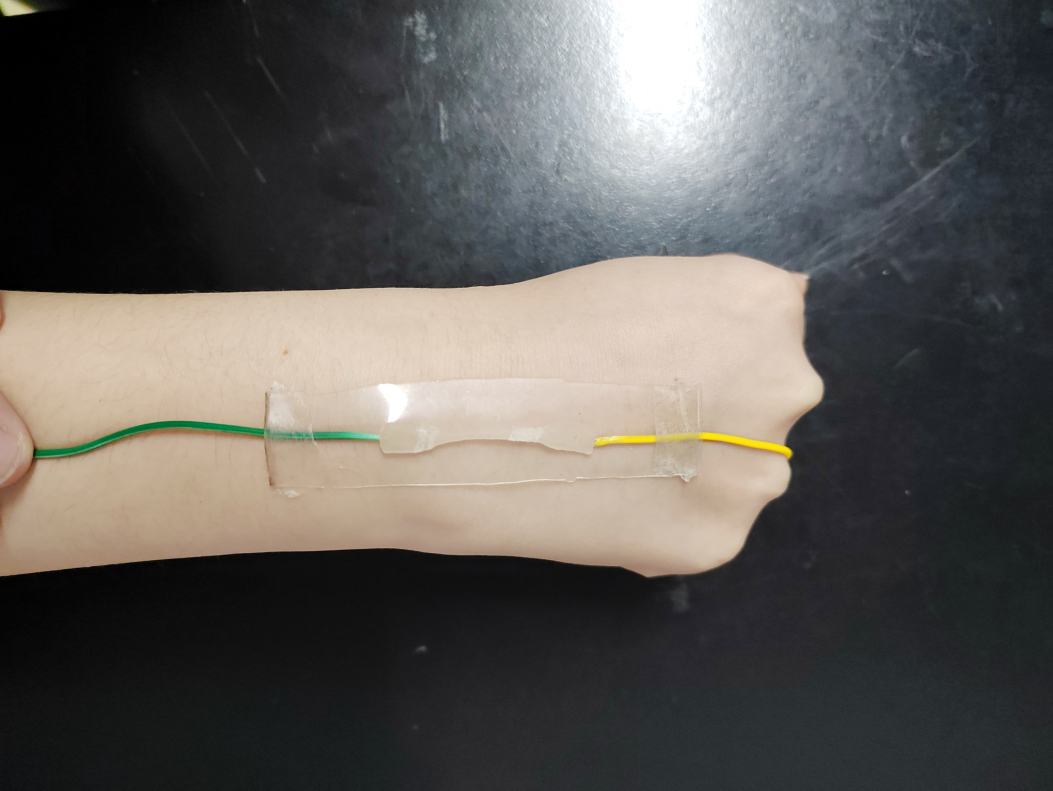


**Figure S17.** Digital photograph of encapsulated sensor based on PXL attached to human wrist for motion test.


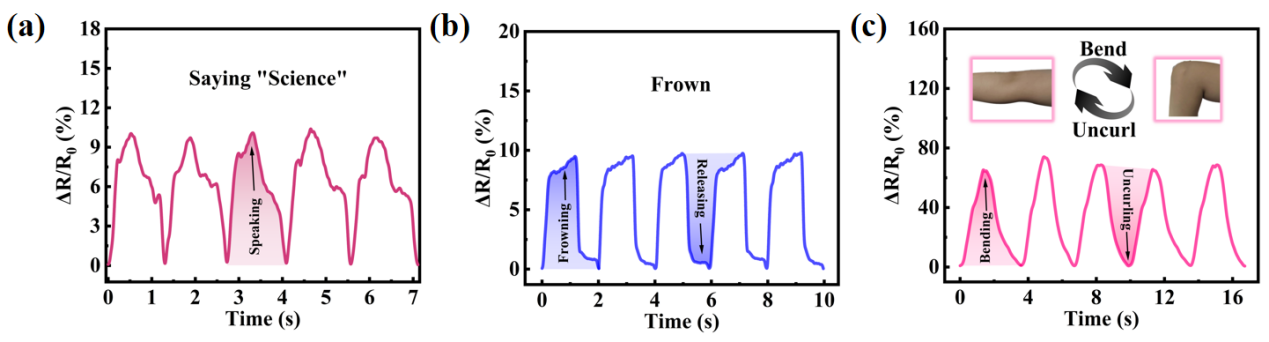


**Figure S18.** Sensing performance test of PXL hydrogel after being placed at room temperature for 7 days. (a) Say “Science”. (b) Frown. (c) Bend the arm.


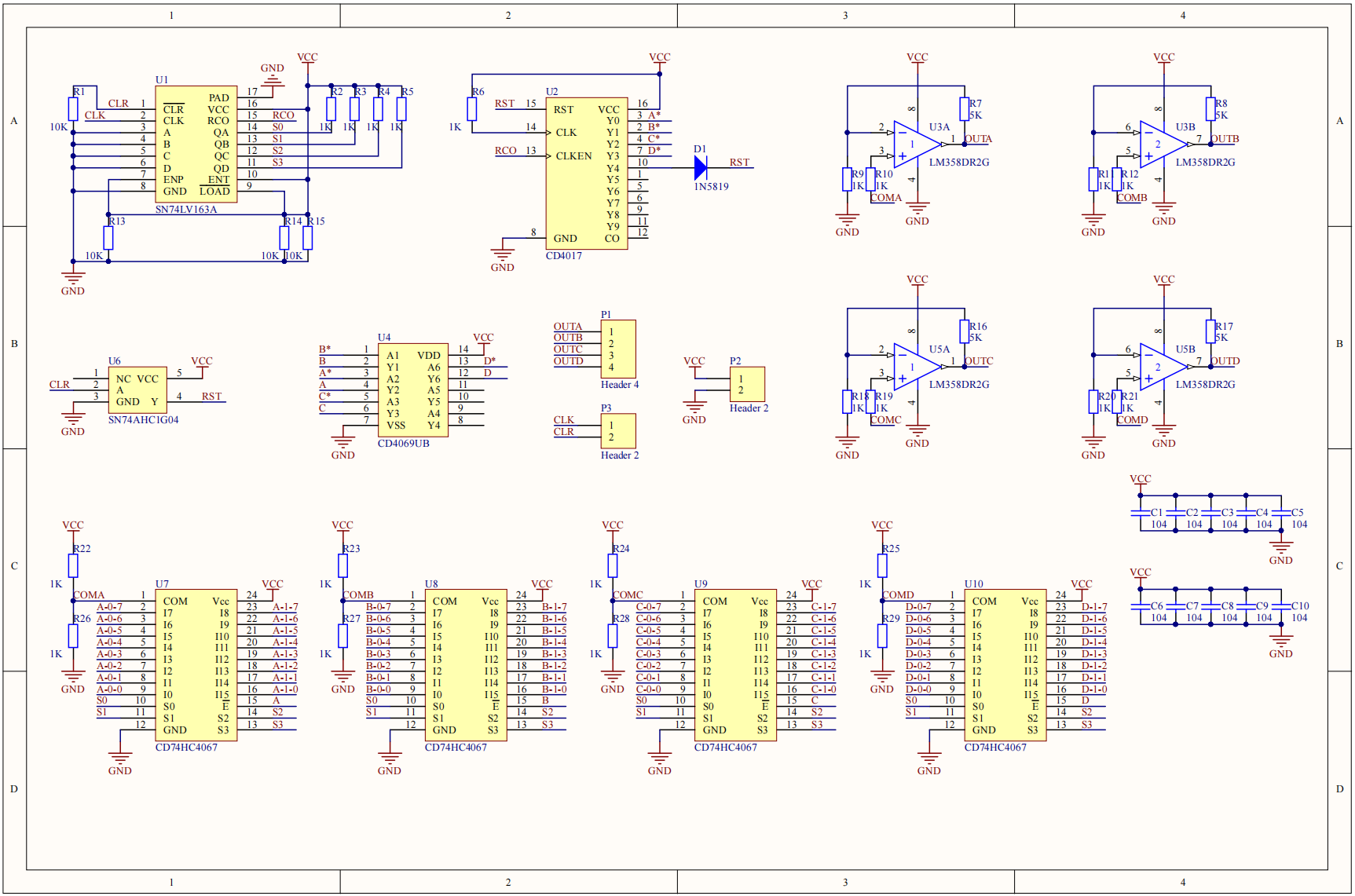


**Figure S19.** Schematic diagram of 8×8 array sensor.

**Experimental Section**

*Materials*: Acrylamide (AM, AR, 99%) was purchased from Shanghai Aladdin Biotech Co., LTD. Xanthan gum (XG, *M*_w_ = 1.1 × 10^6^ g mol^-1^, contained 1.5% pyruvic, viscosity (1% gum in 1% KCl at 20 ℃) is 1484 cps) was purchased from Tianjin Heowns Biochemical Technology Co., LTD. N,N-Methylenebisacrylamide (MBA, 99%) and aluminum chloride (AlCl_3_, 99%) were supplied by Tianjin Damao Chemical Reagent Factory. 2-Hydroxy-4'-(2-hydroxyethoxy)-2-methylpropenone (Irgacure 2959,98%) was provided by Shanghai Macklin Biochemical Technology Co., LTD. Lithium chloride (LiCl, 99%) was purchased from Bidepharm. Urea (99%) was purchased from Tianjin Bohua Chemical Reagent Co., LTD. Polydimethylsiloxane (PDMS) Sylgard 184 purchased from Shenzhen Ausbonds Co., LTD. was used as the packaging elastomer.

*Preparation of Li/Al-LDH Solution*: Li/Al-LDH solution was prepared using urea decomposition method. Specifically, 1 g of LiCl, 3.2 g of AlCl_3_ and 14.4 g of urea were mixed in 100 mL of deionized water and stirred for 15 minutes before refluxed at 97 ℃. A red laser was used to irradiate the solution to track the progress of the reaction. During the reaction process, the Tyndall phenomenon of the solution could be observed, indicating that the size of the LDH nanosheets was between 1 and 100 nm and the LDH nanosheets were evenly distributed. The entire reaction process lasted for 2 hours and ended just before the Tyndall effect of the solution was about to disappear. The Li/Al-LDH solution for preparing the nano-conductive hydrogel PXL was synthesized and used without separation.

*Preparation of PXL Hydrogel*: According to the principle of free radical polymerization, the preparation of PXL hydrogel was divided into two steps, namely: preparation of PXL hydrogel precursor solution and polymerization molding of PXL hydrogel. In the first step, different amounts (i.e., 1, 2, 4, 8 and 10 mL) of freshly prepared Li/Al-LDH solution were first added to the beaker. Then, after making up to 20 mL with deionized water, 3 g of AM monomer and 0.15 g of XG powder were slowly added thereto. The mixture was magnetically stirred for 6 hours and then sonicated in an ice bath for 10 minutes to remove bubbles. Then 9 mg of Irgacure 2959 and 2.4 mg of MBA were added. The mixture was stirred magnetically for 30 minutes and then stood in vacuum for 1 hour to remove oxygen in the solution. Finally, the PXL hydrogel precursor solution was obtained. In the second step, the mixed solution was quickly poured into a polytetrafluoroethylene (PTFE) mold with the desired shape. Then it was tightly covered with a glass sheet to remove bubbles in the mold and isolate air. Finally, polymerization molding was carried out under UV irradiation for 30 minutes to obtain the PXL hydrogel.

*Characterizations*: The particle size of Li/Al-LDH was measured using a laser particle size analyzer (Zetasizer Nano ZEN 3600, Malvern Panalytical, UK). After configuring the refractive indices of Li/Al-LDH and water, the Mie scattering model was employed for the measurements. The electrical conductivity of PXL hydrogel at 5 - 60 ℃ was measured using a four-probe conductivity tester (RTS-11, Tianjin Nuolei Xinda Technology, China), with a probe spacing of 2 mm and a current of 1 mA. The electrochemical impedance spectroscopy (EIS) test of PXL hydrogel was conducted on a Zahner INTERFACE1010E electrochemical workstation (Zahner Elektrik, Germany), using a two-electrode system. Two platinum electrodes were selected as the working electrode and the counter electrode, with a frequency range of 10 mHz to 100 kHz at an open circuit potential with an amplitude of 10 mV. The microstructures of all hydrogels were characterized by FEI company (USA)'s Awavepreo on a field emission high-resolution scanning electron microscope (FE-SEM). Before observation, hydrogels were first freeze-dried, then subjected to a brittle fracture in liquid nitrogen. Subsequently, a thin gold film coating process was applied to avoid charging effects, and finally, the cross-sectional SEM images were observed. The morphological characteristics and particle size of the samples were observed by transmission electron microscope (Thermo Fisher Scientific, USA). A small amount of powder sample was ultrasonically dispersed in ultrapure water/ethanol. 1-2 drops were dropped on a copper grid with a dropper. The floating liquid was absorbed with filter paper. And the float was allowed to stand until dry. Then it was photographed under a 200 kV transmission electron microscope (FEI-Talos F200 S). The chemical composition of the hydrogel was analyzed by Fourier transform infrared spectroscopy (FT-IR) using a VECTOR 22 infrared spectrometer (Bruker, Germany). In the range of 4000-600 cm^-1^, the spectrum was obtained by averaging the signals of 32 scans with a resolution of 4 cm^-1^. Powder X-ray diffraction (XRD) data were collected on a TD-3500 X-ray diffractometer (Dandong, China) at a voltage of 30 kV, a current of 15 mA, a scanning rate of 0.05 ° s^-1^ and a 2θ range of 5° to 80°. The sample was a long strip film with a thickness of about 1 mm.

*Molecular Dynamics Simulation*: The Materials Studio software was utilized to simulate and optimize hydrogels with different compositions and their internal structures. The molecular dynamics (MD) method was employed to simulate the crosslinking process between PAM and MBA within the hydrogels, followed by the prediction of mechanical properties under tensile deformation for the crosslinked hydrogels. All Amorphous Cell models of hydrogels used in the simulations adopted the COMPASSII force field. During the MD simulation of crosslinking, the crosslinking degree was uniformly set to 25% with a time step of 1 fs. The initial equilibration phase utilized an NVT ensemble with a simulation temperature of 298 K, 5,000 steps (5 ps duration). The active dynamics phase employed an NPT ensemble with 25,000 steps (25 ps duration), and trajectories were output every 5,000 steps. Subsequent temperature cycling alternated between NVT and NPT ensembles, with each temperature point running for 20 ps (20,000 steps), temperature increments/decrements of 50 K, maximum energy deviation set to 500,000 kcal mol^-1^, and structural optimization output every 2,000 steps. For the tensile deformation tests, molecular dynamics simulations under an NPT ensemble were performed. Along one principal direction, a strain rate of 5×10^7^ s^-1^ was applied with stepwise incremental stress ranging from 1 to 5 GPa, while maintaining the other two directions at 1 atm and 300 K for 4 ns. Subsequently, this process was repeated for the remaining two directions.

*Mechanical Tests*: The tensile and compressive mechanical properties of hydrogels were measured at 25℃ using a uniaxial material testing system (LIGAO, HF-9002). For tensile tests, all hydrogels used were dumbbell-shaped (3 mm wide and 20 mm long). For compressive tests, all hydrogels used were cylindrical specimens (12 mm in height and 25 mm in diameter).

*Rheological Tests*: The rheological measurement of the hydrogel (with a thickness of 2 mm and a diameter of 10 mm) was tested at room temperature on a rheometer (MARS 60, HAAKE, Germany) with a 20 mm parallel plate at a gap of 3 mm. Amplitude sweeps (strain = 1%) were performed on the hydrogel across 0.1 - 100 rad s^-1^ to measure storage (*G'*) and loss modulus (*G''*).

*Electromechanical Tests*: For tensile sensing, gauge factor (*GF*) was defined by the formula *GF = δ (ΔR/R*_0_*)/δε*, *ΔR/R*_0_ *= (R*_p_*-R*_0_*/R*_0_*)*, where *ε* was strain, *R*_0_ and *R*_p_ corresponded to the resistance at initial and post-tensile (or compressive), respectively. For pressure sensing, the sensitivity (*S*) was defined by the formula *S = δ (ΔR/R*_0_*)/δp*, *ΔR/R*_0_ *= (R*_p_*-R*_0_*/R*_0_*)*, where *P* stood for the pressure. Unless otherwise stated, all experiments were conducted at room temperature (25 °C). Under these conditions, the strain/pressure sensing properties of the PXL hydrogel were tested using a tensile testing machine, computer, and multimeter system.

*Design of Multifunctional Sensors*: The PXL hydrogel sample was sandwiched between two layers of PDMS, fixed at both ends and connected by copper wires to assemble a strain sensor. The sensor was directly attached to volunteers' vocal cords, facial regions, and body joints to monitor human motion, with data recorded in real-time via a LABVIEW-controlled computer interface. It was confirmed that informed written consent from all participants was obtained prior to the experiments with sensors and wearable technologies. The strain sensor was encapsulated with PDMS and did not directly contact human skin. Additionally, only resistance changes of the hydrogel sensor were obtained during the test and no information regarding the human beings was obtained. Therefore, the approval from a national or institutional ethics committee was not required in this case.

*8×8 Array Sensor*: On this basis, in order to make a wearable sensor, an 8×8 sensor array was made to detect the location and change of external pressure. Each pixel was made of a cylindrical hydrogel with a diameter and height of 3 mm. By assembling with a self-designed printed circuit board (PCB), it could generate changes in electrical signals under different pressure stimuli. The electrical signal was recognized, collected and recorded by Arduino, and then transmitted to the computer through the serial port. Finally, the change of pressure was displayed as 64 color blocks of different gray levels through a self-programmed MATLAB program.

*Three-Point Plantar Pressure-Sensing Smart Insole*: In order to fabricate flexible wearable devices, the three-point plantar pressure-sensing smart insole was designed to detect the pressure distribution of the three key parts of the human foot, so as to achieve motion monitoring and disease prevention. The three key plantar sensing parts were all made of cylindrical PXL nano-conductive hydrogel with a height of 5 mm and a diameter of 25 mm. Through the design of a copper wire circuit for assembly with the PXL nano-conductive hydrogel and then using a plastic-encapsulation film with PET as the base material and EVA as the adhesive for plastic encapsulation, the three-point plantar pressure-sensing smart insole was obtained. This smart insole could read the changes in electrical signals generated under different motion states. Then, the STM32 single-chip microcomputer was used to process the read electrical signals, and then the digital signals obtained after processing were transmitted to the mobile terminal through Bluetooth. Finally, the visual monitoring of the real-time plantar pressure distribution was achieved on the self-developed mini-program.

*Statistical Analysis*: The data with an error bar are represented as mean ± standard deviation calculated on a minimum of three independent samples using the Origin software.

**References**

[1] B. Demir, T. R. Walsh, A Robust and Reproducible Procedure for Cross-Linking Thermoset Polymers Using Molecular Simulation, *Soft Matter* **2016**, 12, 2453.

[2] M. An, B. Demir, X. Wan, H. Meng, N. Yang, T. R. Walsh, Predictions of Thermo‐Mechanical Properties of Cross‐Linked Polyacrylamide Hydrogels Using Molecular Simulations, *Adv. Theor. Simul.* **2019**, 2, 1800153.

[3] R. Kumar, A. Parashar, Atomistic Simulations of Pristine and Nanoparticle Reinforced Hydrogels: A Review, *Wiley Interdiscip. Rev. Comput. Mol. Sci.* **2023**, 13, e1655.
